# STRONG prehabilitation before elective ventral hernia repair: Learnings from recruitment challenges in a randomized controlled trial

**DOI:** 10.1371/journal.pone.0360482

**Published:** 2026-10-27

**Authors:** Sofie Anne-Marie Skovbo Jensen, Susanne Vahr Lauridsen, Siv Fonnes, Jacob Rosenberg, Birgitte Brandstrup, Frederik Helgstrand, Hanne Tønnesen

**Affiliations:** 1 WHO-CC, the Parker Institute, Bispebjerg and Frederiksberg Hospital, Frederiksberg, Denmark; 2 Center for Perioperative Optimization, Department of Surgery, Copenhagen University Hospital - Herlev and Gentofte, Herlev, Denmark; 3 Departement of Clinical Medicine, University of Copenhagen, Copenhagen, Denmark; 4 Department of Surgery, Holbæk Hospital, part of Copenhagen University Hospitals, Holbæk, Denmark; 5 Center for Surgical Science, Department of Surgery, Zealand University Hospital, Køge, Denmark; 6 Department of Health Sciences, Faculty of Medicine, Lund University, Lund, Sweden; Faculty of Medicine Vajira Hospital, Navamindradhiraj University, THAILAND

## Abstract

**Background:**

The impact of smoking, nutrition, alcohol, and physical inactivity (SNAP) on the outcome of ventral hernia repair remains underexplored. This trial aimed to evaluate the effect of targeted, intensive lifestyle prehabilitation on postoperative complications.

**Methods:**

The STRONG-Hernia trial (NCT06611462) was a multicenter, parallel-group, centrally randomized controlled trial at two Danish university hospitals. Adults scheduled for elective ventral hernia repair with ≥1 risky SNAP factor were randomized (1:1) to STRONG prehabilitation or standard care. Prehabilitation comprised six weekly in-hospital sessions of education, motivational, and pharmaceutical support tailored to each patient’s SNAP factors. The primary outcome was postoperative complications ≤30 days. Secondary outcomes included lifestyle changes and health-related quality of life (EQ-5D). Final primary outcome assessment and statistical analyses were blinded. Power calculation required inclusion of 2 × 199 patients.

**Results:**

Between March 2024 to May 2025, 43 patients were randomized with 21 to STRONG prehabilitation and 22 to standard care. Around 75% had one risky lifestyle factor, and 25% had ≥ 2; obesity was most common. Shortly after randomization, two prehabilitation participants were excluded due to no surgery. At end-of-intervention, 13/19 in the prehabilitation group had optimized all risky lifestyles versus 0/22 in the control group (p < 0.01). Three additional participants in each group did not undergo surgery. At 30 days, complications occurred in 3/16 prehabilitation and 4/19 control participants (p = 1.00). EQ-5D VAS remained unchanged in the prehabilitation group from baseline but decreased significantly in the control group (p = 0.03). EQ-5D-5L index changes were similar between groups (p = 0.92). Challenges encountered included outsourcing of patients, insufficient recruitment strategy, and a low inclusion rate.

**Conclusion:**

The trial was terminated early due to slow recruitment and was therefore not powered to assess the effect of the prehabilitation intervention on postoperative complications. The findings suggest that the STRONG program seems promising for optimizing risky lifestyles, although these lifestyle results should be considered preliminary. Insights from this trial may help address recruitment challenges essential for completing a future adequately powered trial on postoperative complications.

## Introduction

Ventral hernia repair is a common surgical procedure worldwide, with increasing numbers over the last 20 years, affecting patient quality of life and imposing a significant burden on healthcare systems [1]. In Denmark alone, around 6,000 ventral hernia repairs are performed each year [2], and in the USA, more than 350,000 repairs [3], resulting in considerable costs to the national healthcare systems [3]. Reported complication rates after ventral hernia repair vary widely from 3 to 48% based on small and heterogeneous studies and underpowered randomized controlled trials (RCTs) [4]. Lifestyle factors such as smoking, obesity, alcohol, and physical inactivity have been shown to increase the risk of adverse outcomes after ventral hernia surgery and associated costs [5–7]. These common risky lifestyle factors, also known as the SNAP (Smoking, Nutrition (both obesity and malnutrition), Alcohol, and Physical inactivity) factors, are modifiable and could be targeted through prehabilitation.

Prehabilitation seeks to enhance functional capacity and physiological reserve by addressing modifiable risk factors prior to surgery to improve the patient’s ability to cope with surgical stress, lower complication risks, and support a quicker recovery [8]. However, the effect of multimodal prehabilitation of risky lifestyle factors on postoperative complications is still unclear [9]. Most prehabilitation trials have focused on cancer or major surgery, primarily evaluating the effects of single-component interventions—most commonly preoperative physical exercise—or combinations of two to three elements, typically physical exercise and nutrition [9]. Moreover, there has not been a tendency to tailor all interventions in multimodal prehabilitation to patients’ specific, pre-identified risk factors [10], potentially explaining the inconsistent results observed across trials [9]. Interest in prehabilitation before ventral hernia surgery has also been growing, yet its effectiveness remains uncertain [11]. The evidence is limited, with only two prior RCTs evaluating its impact in this context [12-13]. Thus, the European Hernia Society has called for further research in this area [11].

Our hypothesis was that a targeted, intensive SNAP prehabilitation program would halve the complication rate after surgery compared with standard care. We aimed to investigate the effect of a targeted, intensive SNAP prehabilitation program (STRONG program) compared with standard care on 30-day postoperative complications in patients with at least one risky SNAP factor undergoing ventral hernia repair. Secondly, we aimed to investigate the effect on secondary outcomes, including changes in lifestyle and health-related quality of life (HRQoL).

## Methods

The study was designed as a multicenter, parallel 2-armed, superiority RCT, with an allocation ratio of 1:1. It was registered at www.clinicaltrials.gov (NCT06611462) before recruitment began, and a protocol article was published [14]. The trial was approved by the Danish Scientific Ethical Committee (H-23028872) and the Danish Data Protection Agency (P-2023–14284). The trial was reported according to the CONSORT 2025 statement [15].

### Setting

The trial was originally planned to include three surgical departments in Denmark: Herlev University Hospital, Zealand University Hospital Køge, both part of Copenhagen University Hospitals, and Horsens Regional Hospital. However, Horsens Regional Hospital withdrew shortly before trial initiation as all their patients with ventral hernias were outsourced to the private sector due to long waiting lists. For replacement, the active surgical department at Holbæk Hospital, part of Copenhagen University Hospitals, was opened. At Zealand University Hospital Køge, the trial was closed before recruitment of any participants due to logistic challenges that could not be overcome. The trial was initiated at Herlev Hospital on March 4, 2024. Holbæk Hospital began recruitment of participants on December 1, 2024.

### Eligibility criteria

Inclusion criteria were adults (≥18 years) scheduled for elective ventral hernia repair with a fascia defect smaller than 8 cm and at least one of the following five risky SNAP factors:

- Smoking: Daily use of ≥1 cigarette or 1g tobacco
- Obesity: BMI ≥ 30 kg/m^2^
- Malnutrition: Nutritional Risk Score [16] (NRS) ≥3
- Risky alcohol intake: Over 14 units/week in the past month for both men and women (1 unit = 12g alcohol) [17]
- Physical inactivity: Less than 30 minutes daily or 3.5 hours weekly of physical activity [18]

Originally, only primary ventral hernia repairs (umbilical and epigastric) were included. But the inclusion criteria were quickly expanded to include incisional ventral hernias and all surgical approaches, to enhance the volume of potential participants, after the amendment was approved (May 21, 2024).

Additionally, the anticipated time before surgery had to allow a minimum of four weeks of prehabilitation. Participants were excluded if they had other types of ventral hernias (e.g., para-stomal or hernias with a fascia defect >8 cm), were pregnant or breastfeeding, had allergies or contraindications to the STRONG program’s pharmaceutical, nutritional, or exercise components, or were unable to give informed consent (e.g., under 18, severe mental illness, impaired consciousness, or language barriers). Additional exclusions included a history of alcohol-related delirium or seizures, or withdrawal of consent.

### Randomization and study procedures

The random allocation sequence was created using computer-generated random numbers. It was prepared by an individual who had no other involvement in the trial. The randomization was stratified by hospital, by the number of identified risky SNAP factors (one, two, or more than two), and by the type of hernia (primary vs incisional). Participants were assigned in a 1:1 ratio using variable block sizes from 2 to 6. Randomization and allocation were performed at the baseline visit after participants had provided written informed consent and baseline data collection was completed, and they were carried out by the project personnel responsible for participant enrollment. A computerized, centrally administered “research electronic data capture” (REDCap) system [19] was used for randomization, with the scheme concealed from all project staff. Allocation was not blinded to the project staff who carried out the intervention and follow-ups, including data and outcome collection.

The trained project nurses and the national investigator reviewed all relevant outpatient clinic patient lists for individuals scheduled for surgery at the hospitals. These patients were subsequently screened for eligibility to participate in the study. If eligible, they were provided with both verbal and written information about the study. For those who expressed interest in participating, a visit for inclusion, collection of baseline data, and randomization was scheduled. If allocated to the intervention group, the intervention commenced either right after the randomization at the baseline visit or the nearest possible date. The intervention was delivered by local project nurses or members of the STRONG team at the hospitals where the participants were enrolled. All were trained and certified in the intervention through a 5-day course.

All participants were seen at surgery and three postoperative follow-ups after 1, 3, and 6 months to assess and register any complications and the status of their lifestyles. Blood and urine samples were collected at prespecified follow-ups for study use and for creating a biobank for future research [14].

#### Standard arm.

The control group received standard care at the hospital department where they had been scheduled for surgery. At both Herlev and Holbæk Hospitals, this meant that surgeons typically recommended smoking cessation and weight loss for obese patients at the outpatient visits. However, smoking cessation was not mandatory, nor was it tested before surgery. Surgery was often not scheduled for patients with a BMI > 35, depending on hernia characteristics and symptom severity, but there were no strict criteria for scheduling, deferral, or refraining based on BMI. Participants were free to seek any available lifestyle support outside the project.

#### *Intervention arm*.

In addition to standard care, participants randomized to the intervention group received the STRONG program. It was built on the Gold Standard Program (GSP) for smoking cessation [20] which had been translated to an alcohol cessation program, nutritional program, and physical activity program [14]. Each participant in the intervention group received a tailored program targeting all their risky SNAP factors identified at the screening. It consisted of meetings held once a week for six weeks, generally including follow-up on the status of lifestyle, motivational conversation, education, and optional pharmaceutical support in the preoperative period. The education was closely tied to the participants’ upcoming surgery and covered key areas including an introduction to the STRONG program, risk of complications and exploring motivation and ambivalence, understanding addiction and/or withdrawal symptoms, risks of relapse and management, short- and long-term benefits of lifestyle change strategies for maintaining these changes, and ongoing guidance tailored to their progress in the later stages of the program.

The individual SNAP-based intervention components were as follows. Smokers were offered free, personalized nicotine replacement therapy (NRT) in accordance with participant preferences and nicotine dependency as assessed by Fagerströms Test for Nicotine Dependency [21]. Participants with a risky alcohol intake were offered thiamine (300 mg daily) and combined B-vitamins, chlordiazepoxide for withdrawal symptoms (10 mg), and low-dose disulfiram (200 mg x 2 weekly). Participants who were obese or at risk of malnutrition received a personalized nutritional plan. For obesity, NUPO® meal replacements were also offered for free as an option. Physically inactive participants received a personalized physical exercise plan, including five minutes of inspiratory muscle training daily and other activities amounting to a total of 3.5 hours per week.

Finally, all intervention participants received a logbook to use as a diary at home and for discussion at the weekly intervention meetings, and oral immunonutrition supplements for the final five days before surgery. The intervention was considered completed if the participant had participated in at least five meetings.

### Outcomes

The primary outcome was the number of participants with complications within 30 days, defined as complications requiring treatment. The initial assessment was conducted by trained project nurses or a medical doctor (SASJ) at postoperative follow-ups, by reviewing medical records, and a subsequent interview with the patients. After the study was completed, independent senior surgeons (JR + BB), blinded to group allocation and unaffiliated with the surgical department where the patients were treated, reviewed both medical records and the initial outcome assessment, and determined the primary outcome.

Secondary surgical outcomes included postoperative complications at 90 days and 6 months, Comprehensive Complication Index (CCI) [22], hernia recurrence and reoperation for recurrence, readmission, total length of stay (LOS), visits to primary care, and time back to work/usual activities. Changes in lifestyle outcomes included quitting/optimizing of all, any improvement of any, and quitting/optimizing of separate risky SNAP lifestyles. Quitting/optimizing of the individual lifestyle factors was defined as follows:

- Smoking: no use of any tobacco products (validated by carbon monoxide breath testing)
- Malnutrition: NRS < 3
- Obesity: BMI < 30 or 5–10% weight loss of baseline weight (without developing malnutrition [14]) at the end of intervention and surgery, and <1% gain of body mass (in fat) at 30 days postoperatively. Bioimpedance measurements were used to assess body composition and distinguish fat loss from loss of lean mass
- Alcohol: weekly alcohol intake of zero units at the end of intervention and surgery, and <14 units postoperatively
- Physical inactivity: physical activity >3.5 hours/week

Additional lifestyle outcomes were change in ASA score due to change in lifestyle and change in frailty score. Health outcomes included Health Related Quality of Life (HRQoL). Definitions and time points of all secondary outcomes can be seen in S1 Table.

### Sample size

A sample size was calculated with a two-sided alpha of 5% and a power of 1-beta = 80%. The anticipated complication rate in the control group was set at 20% [14]. The anticipated effect of the prehabilitation program was set at 50%, corresponding to an anticipated complication rate of 10% in the intervention group. The rationale for the anticipated complication rates was detailed in the study protocol [14]. The calculation suggested 199 participants needed in each group to detect a significant risk difference. We aimed to include 2 x 200 participants in the trial. A blinded interim analysis was planned to assess whether the assumptions underlying the sample size calculation remained valid, due to the substantial uncertainty in the initial parameter estimates. The planned interim analysis would also inform about potential futility. Criteria for potential premature study termination were pre-specified in the protocol and included, among others, unsatisfactory participant enrolment [14].

### Statistical methods

Numbers and proportions with 95% confidence intervals (CI) were reported for categorical outcomes. Median and range/interquartile range (IQR) were reported for continuous outcomes, as data were not normally distributed. Comparisons between the intervention and control groups were conducted using Fisher’s exact test for categorical outcomes, the Mann-Whitney-U test for continuous unpaired outcomes. Comparison analyses were conducted blinded by a statistician and by modified intention-to-treat (excluding patients who did not undergo surgery). Simple worst-case imputation was used for missing data on lifestyle outcomes [23], back to work/usual activities, and visits to primary care, to prevent overestimating the results. Statistical significance was defined as a two-sided p-value <0.05. Due to the small number of included patients, we chose not to conduct statistical analyses on all pre-specified secondary outcomes in the protocol [14].

## Results

The trial was terminated before reaching the calculated sample size due to slow patient enrolment. The last participant was recruited in May 2025. In total, 43 participants were enrolled (approximately 11% of the planned sample size of 400 patients), including 35 from Herlev Hospital over 15 months and 7 from Holbæk Hospital over 6 months. The last 30 days postoperative follow-up was conducted on October 2^nd^, 2025. Within the recruitment period from March 2024 to May 2025, 328 patients were scheduled for ventral hernia repair at the two recruiting departments and screened for eligibility. Among these, 118 patients were eligible and invited to participate, and 43 accepted (Fig 1). At randomization, 21 were allocated to the intervention group receiving prehabilitation through the STRONG program, and 22 to the control group receiving standard care.

**Fig 1:**
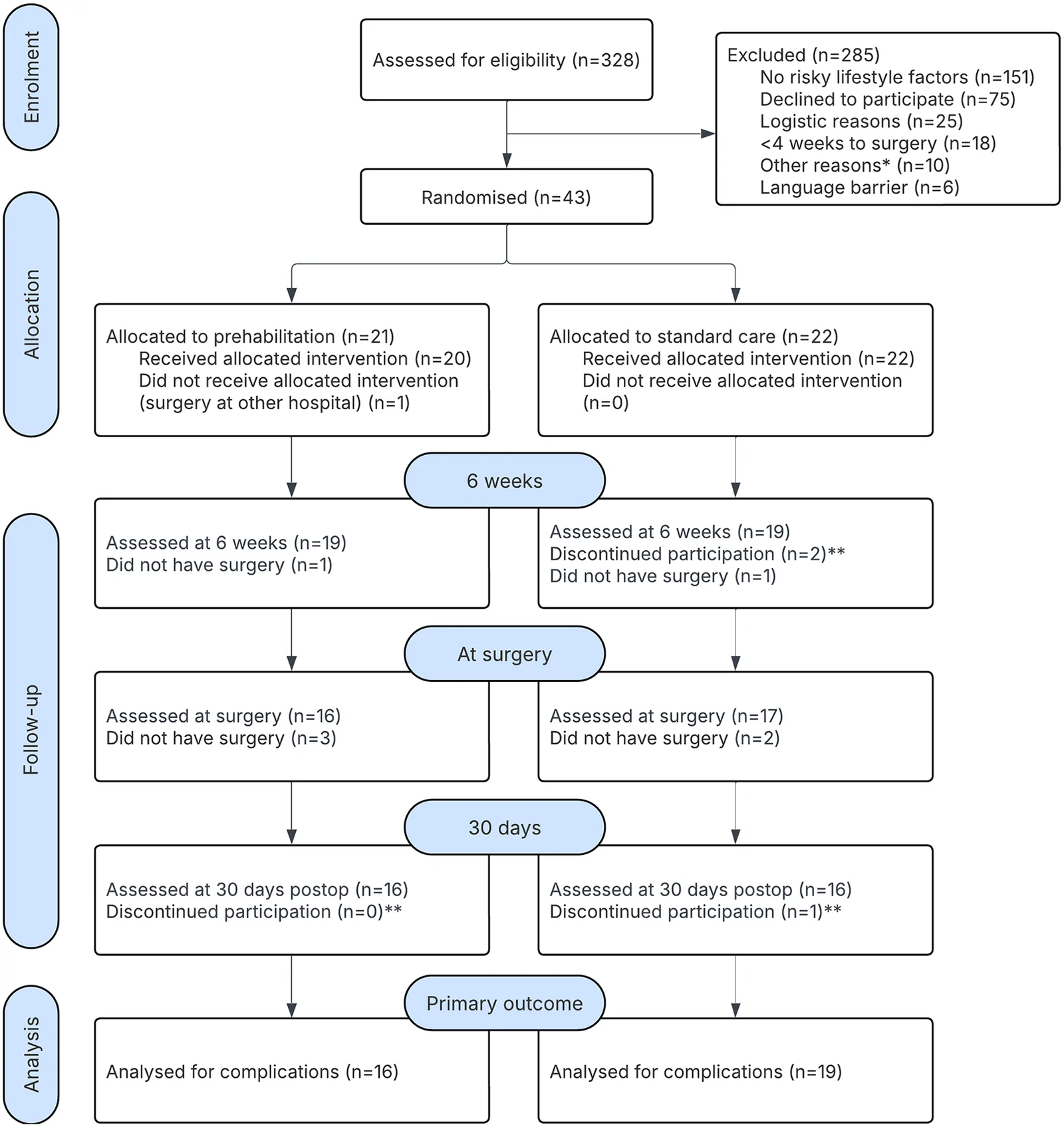
CONSORT flow diagram. *Other reasons cover: severe mental disorder (n = 3), declined to answer questions at screening (n = 2), unstable psychiatric disease (n = 1), previous withdrawal seizures or delirium tremens (n = 1), and laparoscopic/robotic surgery (n = 2) and incisional hernia (n = 1) before inclusion criteria were expanded to cover these. ** Discontinued participation refers to patients who declined further study visits but did not withdraw consent for follow-up through medical record review. Thus, they were available for medical record-based primary outcome assessment.

Among the randomized patients, median age was 64 (IQR 49–67) in the prehabilitation group and 61 (IQR 47–68) in the control group. Groups were comparable regarding the type of hernia and number of SNAP factors, with approximately 75% having one SNAP factor and around 25% having two or three (Table 1). A BMI above 30 was the main risky lifestyle, present in around 70% of both groups. Of these, 13% in the prehabilitation group and 27% in the control group were already using weight loss interventions at the time of inclusion, including GLP-1 analogues, gastric sleeve surgery within six months, or a very low-calorie diet. Median time between baseline and surgery was 96 (IQR 61–123) and 81 (44–108) days in the prehabilitation group and the control group, respectively. There were no harms in any of the groups during the study period. As the study ended prematurely, this report includes outcomes up to 30 days postoperative follow-up.

**Table 1:** Baseline characteristics.

Characteristics	Prehabilitation (n = 21)	Standard care (n = 22)
**Demographics**		
Age, median (IQR)	64 (49–67)	61 (47–68)
Men, n (%)	14 (67)	19 (86)
Primary ventral hernia, n (%)	16 (76)	16 (73)
Education: short or none^a^, n (%)	1 (5)	2 (9)
Employed, n (%)	9 (43)	12 (55)
Living alone, n (%)	8 (38)	9 (41)
Rented accommodation, n (%)	7 (33)	7 (32)
**Lifestyle**		
Smokers, n (%)	2 (10)	6 (27)
Body mass index ≥30.0, n (%)	15 (71)	15 (68)
At risk of malnutrition (NRS ≥ 3), n (%)	2 (10)	0 (0)
Alcohol use > 14 units/week, n (%)	3 (14)	4 (18)
Physical activity <3.5 hours/week, n (%)	5 (24)	4(18)
Number of risky SNAP factors, n (%)		
1	16 (76)	17 (77)
2	4 (19)	3 (14)
3	1 (5)	2 (9)
**Other**		
ASA status, n (%)		
1	1 (5)	0 (0)
2	14 (67)	15 (68)
3	6 (30)	7 (32)
Diabetes type 2, n (%)	2 (10)	3 (14)
Charlson comorbidity score, median (IQR)	2 (1 –3)	2 (0–4)
Fried’s Modified Frailty Score [24], n (%)^b^		
0	8 (42)	10 (45)
1	6 (32)	10 (45)
2	3 (16)	2 (9)
3	2 (11)	0 (0)
Grip strength, kg force, median (IQR)^b^	36 (29–47)	45 (37 –53)
15 foot walking test [32], seconds, median (IQR)^c^	4 (3–4)	4 (3–4)
Metabolic status^d^		
Hemoglobin, median (IQR), mmol/L	8.8 (8.6–9.6)	9.2 (8.7–9.7)
Albumin, median (IQR), g/L	43 (42–44)	41 (38–43)
Bilirubin, median (IQR), µmol/L	9 (9 –12)	9 (7 –16)
ASAT, median (IQR), U/L	21 (17 –29)	21 (15 –27)

^a^: Short or no education level = no education except primary school, high school or short work-related courses. ^b^: Missing data for two participants in the prehabilitation group. ^c^: Missing data for one participant in the prehabilitation group. ^d^: In the prehabilitation group, data for all four blood biomarkers are missing for two participants, and ASAT data are missing for a third participant. g: gram, IQR: interquartile range, L: liter, n: number, SNAP: smoking, nutrition (malnutrition and obesity), alcohol, physical inactivity.

### Surgical outcomes

In the prehabilitation group, five participants did not undergo surgery, with three attributing this to reduced hernia-related discomfort following weight loss. In the control group, three participants did not undergo surgery for health and private reasons. Thus, 16 prehabilitation and 19 control participants underwent surgery (surgical characteristics in S2 Table) and could be analyzed by modified Intention-to-treat analysis for surgical outcomes.

Within the first 30 days, 3/16 (19%, 95% CI 4–46%) participants in the prehabilitation group and 4/19 (21%, 95% CI 6–46%) participants in the control group experienced complications requiring treatment (p = 1.00). Most complications were Clavien–Dindo grade I–II and primarily consisted of surgical site infections and hospital revisits due to pain requiring treatment. One patient in the control group, however, required multiple reoperations (Clavien-Dindo grad IIIb). The median Comprehensive Complication Index was 0 in both groups (range 0–21 and 0–48, respectively), p = 0.78, see Table 2.

**Table 2:** Surgical outcomes at 30 days postoperatively.

Surgical outcomes at 30 days	Prehabilitation group (n = 16)	Control group (n = 19)
	n (%)	n (%)
Patients with complications	3 (19)	4 (21)
Clavien Dindo grade		
I	2 (13)	1 (5)
II	1 (6)	2 (11)
IIIa	0 (0)	0 (0)
IIIb	0 (0)	1 (5)
Type of complication		
Wound complications		
Superficial surgical site infection	1 (6)	2 (11)
Deep surgical site infection	0 (0)	1 (5)
Fascial dehiscence	0 (0)	1 (5)
Other wound complications	1 (6)	0 (0)
Deep surgical site infection	0 (0)	1 (5)
Fascial dehiscence	0 (0)	1 (5)
Other wound complications	1 (6)	0 (0)
Treatment-requiring pain	1 (6)	2 (11)
Reoperation	0 (0)	1 (5)
Seroma	3 (19)	2 (11)
Visits to primary care	10 (63)	15^a^ (79)
Back to work or usual activities	11 (69)	12^b^ (63)

^a^: four had missing data and were considered as worst-case scenarios, i.e., had at least one visit to primary care. ^b^: three had missing data and were considered as worst-case scenarios, i.e., not returned to work/usual activities.

Median total postoperative length of stay within 30 days was 5 hours (IQR 3–19, range 1–60) in the prehabilitation group and 5 hours (IQR 4–15, range 2–203) in the control group. In total, 63% (95% CI 35–85%) and 79% (95% CI 54–94%) had at least one visit to primary care, typically for the removal of stitches, and finally, 69% (95% CI 41–89%) and 63% (95% CI 38–84%) had returned to work or usual activities, respectively. No participants in either group experienced hernia recurrence during the 30-day follow-up period. A more detailed description of surgical outcomes at 30 days is presented in Table 2.

### Change in lifestyle outcomes

At the end of the intervention, 19 and 20 participants in the prehabilitation and control groups, respectively, were still scheduled for surgery. In the prehabilitation group, the adherence to intervention meetings was high: 18/19 participated in ≥ five meetings.

At the end of intervention, 68% (95% CI 43–87%) in the prehabilitation group and 0% (95% CI 0–17%) in the control group had optimized all their risky SNAP lifestyles (p < 0.001; Table 3). A total of 95% (95% CI 74–100%) vs. 60% (95% CI 36–81%) of participants had improved at least one risky SNAP lifestyle, respectively. All lifestyle outcomes at the end of intervention are presented in Table 3, except for frailty, for which we refrained from reporting, as only two of the 43 randomized participants were frail at baseline (frailty score ≥3, Table 1).

**Table 3:** Changes in lifestyle outcomes at the end of the intervention.

Outcome	Prehabilitation group			Control group		
	N	n (%)	95% CI	N	n (%)	95% CI
Optimizing all risky lifestyles	19	13 (68)	43–87%	20	0 (0)	0–17%
Improvement in any risky lifestyle	19	18 (95)	74–100%	20	12 (60)	36–81%
Optimizing individual risky lifestyles						
Smoking	1	0 (0)	–	5	0 (0)	–
Obesity	14	11 (79)	–	14^a^	2 (14)	–
Malnutrition	1	1 (100)	–	0	–	–
Alcohol	3	2 (67)	–	4	0 (0)	–
Physical inactivity	4	3 (75)	–	4	1 (25)	–
Change in ASA score	19	2 (11)	1–33%	20	3 (15)	3–38%

^a^One additional participant was obese but underwent surgery within four weeks of inclusion and was therefore excluded at this time point. N: number of participants assessed, n: number of participants achieving the outcome.

Results for all lifestyle outcomes at surgery and 30 days postoperatively are presented in S3 Table. Optimizing all risky SNAP lifestyles over time is presented in Fig 2. The frequency of participants who optimized all risky lifestyles diminished slowly over time in the intervention group, while a few additional participants optimized all risky lifestyles over time in the control group.

**Fig 2:**
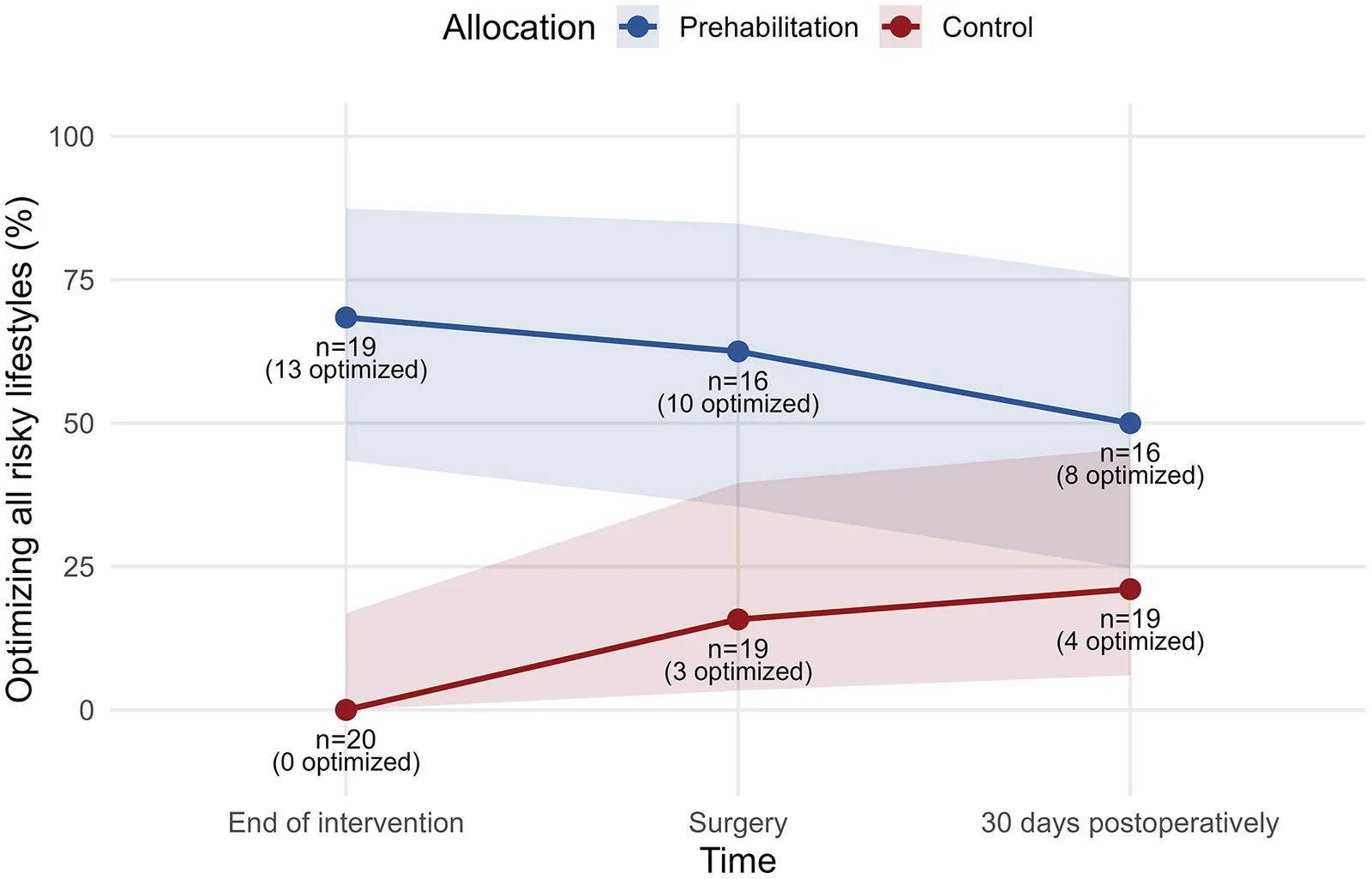
Optimizing all risky SNAP lifestyles over time. Shaded areas indicate 95% confidence intervals. n = number of total participants in the group.

### Change in health-related quality of life

HRQoL was comparable at baseline between the prehabilitation and control groups. Individual changes from baseline to 30 days postoperatively varied widely in both groups. Median change in EQ-5D VAS was 0 (range −20–62) in the prehabilitation group and −15 (range −45–10) in the control group, with a significantly greater decrease in the control group (p = 0.03). Median change in EQ-5D-5L was 0.04 (range −0.03–0.21) and 0.05 (range −0.19–0.41), respectively (p = 0.92). Median EQ-5D VAS and EQ-5D-5L Indexes at different time points in the two groups are presented in S1 Fig.

### Other analyses

Parametric results for continuous outcomes are presented in S4 Table to benefit future meta-analyses.

## Discussion

The study had to be terminated before full inclusion due to slow recruitment, as only 43 of the 400 planned patients were recruited over 14 months. Participants in the STRONG prehabilitation group showed significantly greater success in optimizing all preoperative risky lifestyle factors than those in the control group, but around one-fifth in each group developed complications requiring treatment within 30 days. In addition, changes in EQ-5D VAS from baseline to 30 days postoperatively significantly favored the STRONG prehabilitation group while changes in EQ-5D-5L index showed no difference between groups.

Although terminated before reaching the planned sample size, this study is reported as a prematurely terminated RCT rather than a pilot study, as it was originally designed, registered, and initiated as a fully powered randomized trial. The analysis on complications between the prehabilitation and standard-care groups was inconclusive due to the limited power. Thus, the primary study hypothesis can neither be confirmed nor rejected and the observed complication rates should be regarded as exploratory rather than evidence of treatment effect. Only a few previous RCTs have evaluated SNAP prehabilitation before ventral hernia repair, and none included all five SNAP risk factors as in our study. An older trial from 2008 reported significantly fewer complications at 21% in the intervention group as compared with 41% in the control group after intensive smoking cessation in a mixed surgical population, including umbilical hernia repair [12]. A more recent trial investigating nutrition and physical activity before ventral hernia repair found a non-significant trend toward fewer 30-day wound complications and initially more hernia- and complication-free patients in the prehabilitation group, although no differences persisted at the two-year follow-up [13,25]. Two small pilot randomized trials of very low–calorie diet prehabilitation in patients with obesity undergoing mixed elective general surgery procedures, including ventral hernia repair, reported postoperative complications in 4/21 vs. 8/22 in one study [26], and wound infections in 0/20 vs 2/20 in the other [27], for the intervention and standard care groups, respectively. Overall, evidence for multimodal SNAP prehabilitation for ventral hernia repair remains limited.

The STRONG prehabilitation intervention was effective in supporting patients to optimize all preoperative risky SNAP lifestyle factors at end-of-intervention. This aligns with the documented positive effects of the Danish Gold Standard Programme (GSP), on which the STRONG program is modelled, on smoking cessation [20] and with a recent RCT showing successful smoking and alcohol cessation with a GSP-based intervention [28]. The STRONG prehabilitation seems promising for preoperative optimization of risky lifestyles. Furthermore, as an interesting observation, three of the fifteen obese patients in the prehabilitation group chose to cancel their surgery, attributing it to reduced hernia-related discomfort following weight loss.

The early trial termination was caused by different challenges, ultimately resulting in slow recruitment. Fewer patients were scheduled for surgery at the recruiting departments than expected, even after expanding the inclusion criteria to all surgical approaches and incisional hernias early in the process. In Denmark, healthcare is publicly funded and provided free of charge, and patients have the right to choose publicly funded surgery at a private hospital if the waiting time exceeds 30 days following the indication for surgery [29]. Throughout the study period, several patients therefore opted for an operation in a private hospital without possibilities for prehabilitation and were not included. One participating center withdrew before trial initiation after diverting all elective hernia repairs to private hospitals. In addition, the long waiting times extended the prehabilitation period well beyond the expected six weeks. While the longer period allowed more time to achieve lifestyle changes, it required participants who were already optimized within six weeks to maintain changes with less support, increasing the risk of relapse and of losing motivation. The first site also experienced a reorganization, which created logistical problems for recruitment, preventing patients from being screened and offered participation face-to-face during their initial and only outpatient visit. As a result, some patients could not be contacted after they had left the clinic after scheduling their hernia repair. In addition, some patients with a BMI above 35 were not scheduled for surgery but were recommended to contact primary care for lifestyle intervention. However, the majority did not return to the outpatient clinic during the study period. It was considered to increase recruitment by involving private clinics or additional public hospitals [14], but many of the logistical challenges would likely have applied there as well.

Finally, the inclusion rate was low, with only 36% of invited patients consenting to participate. As the standard ventral hernia repair pathway typically includes only one preoperative visit for surgical scheduling and no planned postoperative control visits, the intervention and follow-up visits could not be coordinated with pre-scheduled appointments, requiring additional visits solely for trial participation. From a trial-related interview study, we learned that patients who declined, found participation time-consuming. Furthermore, they expressed ambivalence regarding their need for lifestyle improvement, the extent of personal benefit from participating, and whether they could prioritize the required time [30]. This may suggest a potential strategy of closer dialogue with eligible participants about the aim and benefits of the prehabilitation.

Using a multi-faceted approach with a broader recruitment platform, including both the hospital setting and the press/social media for direct recruitment of the patients already scheduled for surgery, may bypass some of the challenges. In addition, concentrating the patient information, screening, randomization, delivery of prehabilitation, follow-up, and coordination at one central outpatient clinic exclusively may reduce the time consumption for the prehabilitation staff. However, this centralized model may introduce selection bias, as participation is more likely among highly motivated patients and those living closer to the center, potentially limiting the inclusion of more vulnerable patients with greater needs. Reducing the number of required visits might further facilitate participation. However, evidence from smoking cessation research has shown that less intensive interventions have reduced quit rates [31], and had no effect on postoperative complications [32]. We learned that changes to the recruitment strategy need to be made and tested in a feasibility study before a future sizable randomized controlled trial is conducted.

### Strengths and limitations

Strengths of this study include the RCT design, which provides the highest level of evidence for causal inference. Secondly, prehabilitation was offered exclusively to patients with one or more identified risky lifestyles at screening, ensuring a targeted intervention and allowing the effect to be estimated specifically among those at risk. The program addressed all five risky SNAP factors, and all intervention providers completed a three-day course in the Danish Gold Standard Program for smoking cessation and an additional three-day, trial-specific course in the intensive STRONG multimodal SNAP prehabilitation. Although primary outcome data were collected by non-blinded project staff, the primary outcome assessment was performed by independent senior surgeons who were blinded to allocation, reducing the risk of detection bias. Finally, adherence to intervention visits was high, indicating that the prehabilitation was meaningful to the participants. Several limitations also apply. The early termination resulted in a small sample size and insufficient power, making the analysis on postoperative complications exploratory, but it also highlights the complexity and difficulties in making a comprehensive RCT. Secondly, generalizability may be limited to other minor surgical procedures involving patient populations like those undergoing ventral hernia repair, rather than to all surgical settings. The low acceptance rate of 36% may have introduced selection bias, as patients who accepted participation may have been more highly motivated than those who declined, thereby reducing the transferability to clinical practice. Participation in an RCT may also have prompted lifestyle changes in the control group, meaning they may not fully reflect standard care, potentially diminishing the observable effect of prehabilitation. Furthermore, the effect of prehabilitation was not evaluated in many patients with a BMI > 35, as they were often not referred for surgery. This limits generalizability to a group for whom it would be particularly valuable to know whether prehabilitation could improve outcomes enough to make surgery feasible. Finally, postoperative complications may have been underestimated in the control group, as 4 of 19 control participants did not attend the 30-day evaluation and were assessed only through medical records, which may have missed complications managed outside the regional system, abroad, or in primary care (e.g., antibiotics for wound infections).

### Perspectives

From the patient’s perspective, avoiding short-term postoperative complications and achieving better long-term health are important. To support this, preoperative information and optimization should be based on solid evidence, which is sparse today regarding prehabilitation before ventral hernia repair. From the clinical perspective, there is a need to establish and implement evidence-based guidelines in this field. Because lifestyle changes can be difficult for many patients, intensive and structured programs like STRONG may be necessary. The high volume of ventral hernia repair makes it clinically relevant for surgical departments and clinics to participate in RCTs. For society, improving public health and reducing the economic burden associated with short-term postoperative complications and long-term persistent risky lifestyles is attractive. From a research perspective, it is relevant to close the current knowledge gap through large, well-planned and well-conducted trials, preceded by minor feasibility studies to adjust for experienced challenges. Future trials may consider focusing on patients with more severe SNAP-related risk profiles, which could improve statistical power for detecting differences in complications. Furthermore, the recruitment strategy needs to be redesigned. Future studies may also investigate whether a longer period between enrolment and surgery allows greater achievement and maintenance of lifestyle changes. Furthermore, long-term follow-up of outcomes such as sustained lifestyle change, quality of life, and healthcare utilization may provide additional insight into the broader effects of prehabilitation. Objective verification of lifestyle changes through biomarkers and body composition measurements should continue to be incorporated whenever feasible. Until sufficient evidence exists, collecting prospective data on risky lifestyle factors and outcomes is important to facilitate high-quality observational studies.

## Conclusion

This trial was terminated early due to slow recruitment and was therefore underpowered to assess the effect of the prehabilitation intervention on postoperative complications. The STRONG prehabilitation intervention seemed to effectively help participants optimize their preoperative risky lifestyles compared to the control group, although these lifestyle results should be considered preliminary. Among the 35 patients who underwent surgery, around one in five in each group developed postoperative complications. The challenges encountered and the lessons learned from this trial may help inform improvements in logistics and recruitment strategies for a future adequately powered trial on SNAP prehabilitation before ventral hernia repair.

## Supporting information

S1 Table Overview of reported outcomes. (PDF)

S2 Table Surgical characteristics. (PDF)

S3 Table Additional changes in lifestyle outcomes. (PDF)

S4 Table Parametric results for continuous outcome variables. (PDF)

S1 Fig Median EQ-5D VAS and EQ-5D-5L index scores over time. (PDF)

S1 File CONSORT Checklist. (PDF)

S2 File Protocol approved by ethics committee. (PDF)
