# Supplementary material for: STRONG prehabilitation before elective ventral hernia repair: Learnings from recruitment challenges in a randomized controlled trial
Source: PLoS One. 2026 Oct 27;21(10):e0360482. doi: 10.1371/journal.pone.0360482 (PMC13712517; doi:10.1371/journal.pone.0360482)
Supplement: Supplementary file 1 [file pone.0360482.s001.pdf]

Supplemental Material

S1 Table. Overview of reported outcomes.

Remaining planned outcomes from protocol article [15] were not reported in this article.

Outcomes	Definition	Instrument	End of intervention	Surgery	30 days postop.
**Surgical**					
Complications	Number of participants with at least one complication, defined as requiring treatment	Medical records, interview and clinical examination. Subsequent review of the assessment against medical records by independent senior surgeons.			X
CCI [33]	Calculated from the Clavien-Dindo classification of the postoperative complications	Medical records and interview			X
Hernia recurrence	Number of participants with recurrence	Clinical examination and interview			X
Reoperation for hernia recurrence	Number of participants with reoperation for recurrence	Medical records and interview			X
Total length of stay	Total time spend in-hospital from the end of surgery and within the first 30 postoperative days	Medical records and interview			X
Return to work/usual activities	Number of patients either returned to work or usual activities[a]	Interview			X
Primary care visits	Number of participants with at least one visit to primary care	Interview			X
**Changes in lifestyle**					
Quitting/optimizing all risky lifestyles	Number of participants who quit/optimized all risky lifestyles identified at baseline. See protocol for definitions of quitting/optimizing a lifestyle [15]	Interview and measurements	X	X	X
Any improvement in any risky lifestyles	Number of participants who made any improvement in any of their risky lifestyles identified at baseline. See protocol for definitions of any improvement in the different risky lifestyles [15]	Interview and measurements	X	X	X
Quitting/optimizing separate risky lifestyles	Number of participants who quit/optimized the separate risky lifestyles identified at baseline.	Interview and measurements	X	X	X

Change in ASA score	Number of participants with improvement in ASA score [34] compared with baseline and related to an improvement in risky lifestyles	Medical records and interview	X	X	X
Change in frailty score	Number of participants with improvement in Fried's Modified Frailty Score [32] compared with baseline	Measurements and interview	X	X	X
**Health**					
HRQoL	Change from baseline	EQ-5D [35]	X	X	X

[a]: original definition in protocol was number of days/months. Changed due to premature ending with reporting limited to 30 days postoperatively.
ASA: American Society of Anesthesiologists, CCI: comprehensive complication index, EQ-5D: EuroQol 5 Dimensions, HRQoL: health-related quality of life.

33. Slankamenac K, Graf R, Barkun J, Puhan MA, Clavien PA. The comprehensive complication index: a novel continuous scale to measure surgical morbidity. Ann Surg 2013;258:1–7.

34. American Society of Anesthesiologists Statement on ASA Physical Status Classification System. Anesthesiology Open 2026;1:pe0002.

35. Stolk E, Ludwig K, Rand K, van Hout B, Ramos-Goñi JM. Overview, update, and lessons learned from the International EQ-5D-5L Valuation Work: version 2 of the EQ-5D-5L valuation protocol. Value Health 2019;22:23–30.
