# Supplementary material for: STRONG prehabilitation before elective ventral hernia repair: Learnings from recruitment challenges in a randomized controlled trial
Source: PLoS One. 2026 Oct 27;21(10):e0360482. doi: 10.1371/journal.pone.0360482 (PMC13712517; doi:10.1371/journal.pone.0360482)
Supplement: Supplementary file 2 [file pone.0360482.s002.pdf]

S2 Table. Surgical characteristics

	Prehabilitation (n = 16)	Standard care (n = 19)
Type of hernia, n (%)		
Primary ventral hernia repair	12 (75)	14 (74)
Incisional hernia repair	4 (25)	5 (26)
Hernia defect width, cm, median (IQR)	2 (2–4)	4 (3–5)
Surgical approach, n (%)		
Open	9 (56)	10 (53)
Laparoscopic*	7 (44)	9 (47)
Mesh placement, n (%)		
Onlay	7 (44)	5 (26)
Preperitoneal	5 (31)	7 (37)
Retromuscular	3 (19)	2 (11)
Intraperitoneal	0 (0)	1 (5)
Other/unknown	0 (0)	3 (16)
Suture only	1 (6)	1 (5)
Concommitant inguinal repair, n (%)	1 (6)	1 (5)
Duration of surgery, minutes, median (IQR)	155 (46–114)	86 (52–91)

All numbers are rounded to the nearest whole number. *: conventional or robotic, n: number.
