# Supplementary material for: STRONG prehabilitation before elective ventral hernia repair: Learnings from recruitment challenges in a randomized controlled trial
Source: PLoS One. 2026 Oct 27;21(10):e0360482. doi: 10.1371/journal.pone.0360482 (PMC13712517; doi:10.1371/journal.pone.0360482)
Supplement: Supplementary file 3 [file pone.0360482.s003.pdf]

S3 Table. Additional changes in lifestyle outcomes

Changes in lifestyles	Prehabilitation group			Control group		
	N	n (%)	95% CI	N	n (%)	95% CI
**At surgery**						
Optimizing all risky lifestyles	16	10 (63)	35–85%	19	3 (16)	3–40%
Improvement in any risky lifestyles	16	13 (81)	54–96%	19	13 (69)	43–87%
Optimizing individual lifestyles						
Smoking	1	0 (0)		4	0 (0)	
Obesity	11	7 (64)		13	2 (15)	
Malnutrition	1	1 (100)		0	–	
Alcohol	3	2 (67)		4	2 (50)	
Physical inactivity	3	3 (100)		3	1 (33)	
Change in ASA score	16	1 (6)	0–30%	19	4 (21)	6–46%
**At 30 days postoperatively**						
Optimizing all risky lifestyles	16	8 (50)	25–75%	19	4 (21)	6–46%
Improvement in any risky lifestyles	16	13 (81)	54–96%	19	10 (53)	29–76%
Optimizing individual lifestyles						
Smoking	1	0 (0)		4	0 (0)	
Obesity	11	7 (64)		13	2 (15)	
Malnutrition	1	1 (100)		0	–	
Alcohol	3	1 (33)		4	2 (50)	
Physical inactivity	3	2 (67)		3	2 (67)	
Change in ASA score	16	1 (6)	0–30%	19	3 (16)	3–40%

CI: confidence interval, N: number of participants assessed, n: number of participants achieving the outcome.
