# Supplementary material for: STRONG prehabilitation before elective ventral hernia repair: Learnings from recruitment challenges in a randomized controlled trial
Source: PLoS One. 2026 Oct 27;21(10):e0360482. doi: 10.1371/journal.pone.0360482 (PMC13712517; doi:10.1371/journal.pone.0360482)
Supplement: Supplementary file 4 [file pone.0360482.s004.pdf]

S4 Table. Parametric results for continuous outcome variables

	Baseline				End-of-intervention				Surgery				30 days postop.			
**Outcomes**	**P**		**C**		**P**		**C**		**P**		**C**		**P**		**C**	
**Surgical**																
CCI, mean ±SD													2	±6	5	±12
Total length of stay, hours, mean ±SD													13	±15	24	±49
**Health**																
EQ-5D VAS, mean ±SD	73	±17	73	±20	85	±11	75	±17	84	±12	74	±16	80	±16	70	±19
EQ-5D-5L Index, mean ±SD	0.85	±0.16	0.84	±0.16	0.88	±0.15	0.89	±0.10	0.91	±0.08	0.86	±0.15	0.93	±0.09	0.87	±0.16

C: control group, CCI: Comprehensive Complication Index, P: prehabilitation group, postop: postoperatively, SD: standard deviation
