# Supplementary material for: STRONG prehabilitation before elective ventral hernia repair: Learnings from recruitment challenges in a randomized controlled trial
Source: PLoS One. 2026 Oct 27;21(10):e0360482. doi: 10.1371/journal.pone.0360482 (PMC13712517; doi:10.1371/journal.pone.0360482)

S1 Figure. Median EQ-5D VAS and EQ-5D-5L index scores over time. Health-Related Quality of Life outcomes are presented as A) median EQ-5D VAS scores and B) median EQ-5D-5L index scores over time with shaded areas indicating interquartile ranges (IQR). Medians and IQR were calculated based on available data. The number of participants with available data from the prehabilitation and control group, respectively, was 21 and 22 at baseline, 18 and 16 at end-of-intervention, 16 and 16 at surgery, and 16 and 15 at 30 days postoperatively.

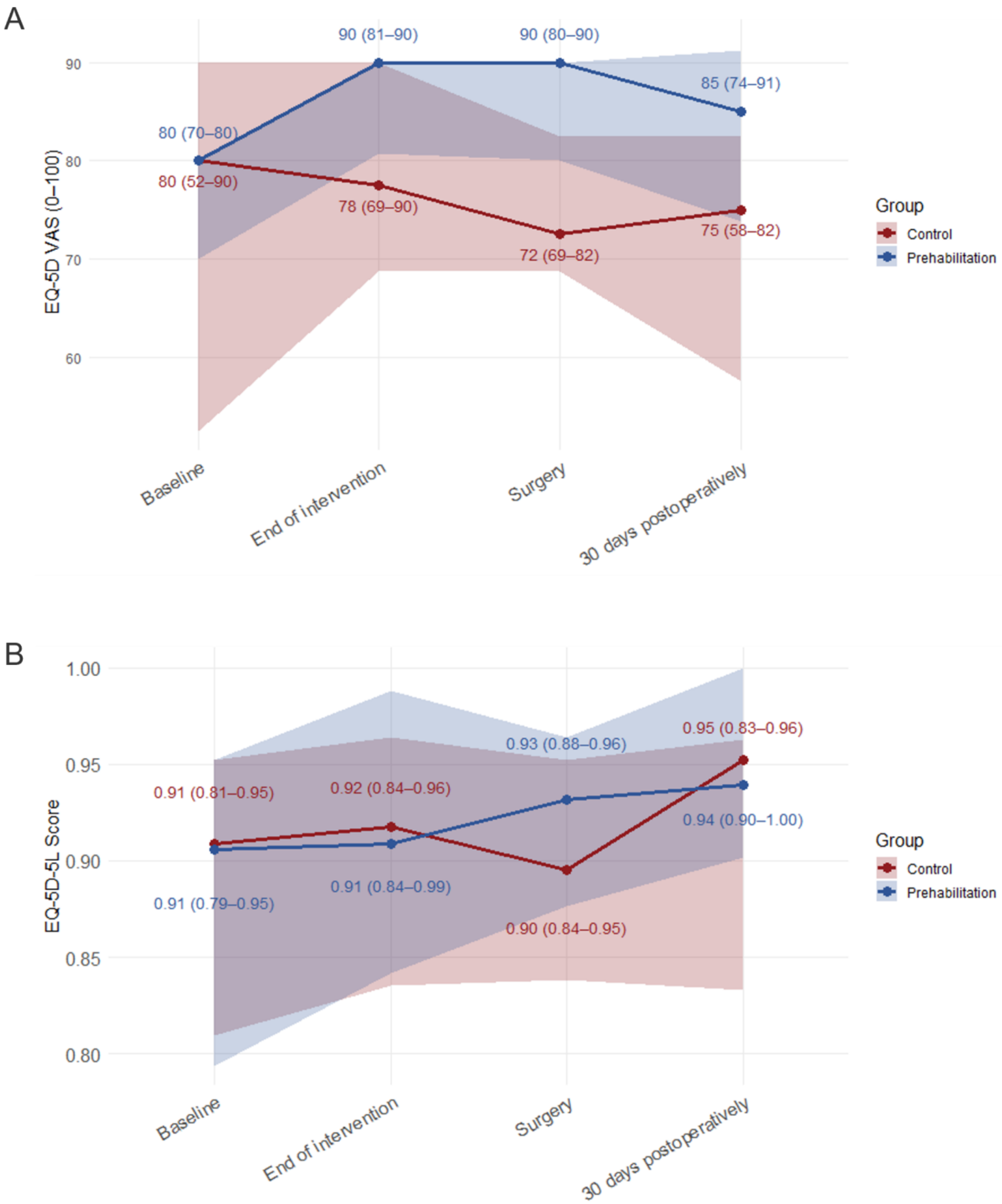

Supplement: Supplementary file 5 [file pone.0360482.s005.pdf]
