# Supplementary material for: STRONG prehabilitation before elective ventral hernia repair: Learnings from recruitment challenges in a randomized controlled trial
Source: PLoS One. 2026 Oct 27;21(10):e0360482. doi: 10.1371/journal.pone.0360482 (PMC13712517; doi:10.1371/journal.pone.0360482)
Supplement: Supplementary file 7 [file pone.0360482.s007.pdf]

S2 File. Protocol approved by ethics committee

1. Title: STRONG FOR SURGERY - STRONG FOR LIFE (STRONG-Hernia)

STRONG prehabilitation for risk reduction prior to ventral hernia repair: Postoperative complications, health, and costs on short & longer term

Præhabilitering for risikoreduktion inden brokoperation: postoperative komplikationer, sundhed og omkostninger på kort og længere sigt

Brief Summary (English)

This national project is the first to include all SNAP factors (Smoking, Nutrition - both obesity & malnutrition - Alcohol, and Physical activity) in a combined intervention aiming at risk reduction for patients scheduled for ventral hernia repair. It will generate new knowledge of the effect on risk reduction via intensive lifestyle intervention 4 weeks prior to surgery.

The overall goal is to reduce the inequity in health via perioperative STRONG lifestyle intervention aiming at a better health gain by surgical risk reduction at short time and improved health on longer time. In total, 2x200 surgical patients disadvantaged by preoperative risky lifestyle will participate.

The primary outcome is postoperative complications. Other important outcomes are clinically relevant lifestyle improvements, frailty and comorbidity symptoms, patient motivation and reflections, health-related quality of life and cost-effectiveness on short and longer time. In addition, possible dose responses between lifestyle at surgery and complications, health & costs, and association between self-reported and validated lifestyles.

This project builds further on the ongoing and smaller STRONG-Cancer project for risk reduction at surgery with change in lifestyle as the primary outcome, approved in 2020 (H-20081571; P-2020-95).

Collaboration among:

4 Surgical Departments at:

HERLEV Hospital (Jacob Rosenberg), HORSENS Regional Hospital (Hans Friis-Andersen), Zealand University Hospital KØGE (Frederik Helgstrand), and Holbæk Hospital (Birgitte Brandstrup)

and

The Danish Centre for Health Economics at University of Southern Denmark (Kim Rose Olsen)

and

The STRONG Team at WHO-CC, Parker Institute at Bispebjerg-FREDERIKSBERG Hospital (Hanne Tønnesen)

Table of Content

1. Title: STRONG FOR SURGERY - STRONG FOR LIFE (STRONG-Hernia) 1
2. Objectives 3
2a) Problem description, hypotheses, outcomes, and the rationale 3
2b) The background (Reference numbers refer to the list in chapter 16) 4
2c) STRONG-Hernia is powered for evaluation for postoperative complications 6
3. Methods 7
3a. Designs, framework, analyses, randomisation, and control group 7
The practical procedures, studies, and outcomes 8
3c. Interventions 10
4. Statistical considerations: Power calculation 13
5. Participants and criteria 13
6. Risks, side-effects, and inconvenience 13
6a) Considerations 13
6b) Discontinuation 14
7. Sampling of blood and urine in our research biobank for biomarkers 14
8. Collection of information from the medical records 15
9. Data Handling 15
9a) Handling and storage 15
9b) Organization 15
10. Economy 16
11. Payments for participation 16
12. Recruitment of participants and informed consent 16
13. Dissemination of results 17
14 Scientific Ethical Considerations 17
15. Appendix: Data collection forms as integrated in the Redcap (in Danish) 18
16. Reference list 18

2. Objectives

2a) Problem description, hypotheses, outcomes, and the rationale

Despite the great developments taking place during and after surgery, e.g., intraoperative techniques[1] and enhanced recovery after surgery (the ERAS protocol[2]), there is a large untapped potential for risk reduction in the preoperative period via effective lifestyle intervention targeting one or more co-existing risky lifestyles: Smoking, malNutrition, obesity, risky Alcohol intake and insufficient Physical activity (SNAP)[3].

The STRONG project is national multicentre research with international impact. The overall goal is to obtain the potential extra health gain from the individually tailored, combined and intensive lifestyle intervention – STRONG - in relation to surgery, thereby adding to lower the inequity in health.
As SNAP factors are frequent and have a negative social gradient, they strike hardest among disadvantaged patients, thereby developing most postoperative complications. In this project we would be able to reach out to surgical patients, who have not been reachable otherwise.

This clinical research benefits from mixed methods and comprehends several sub-studies:

1. Efficacy of the 6-week preoperative STRONG-program compared to treatment as usual (TAU) on (RCT):
   A. risky lifestyles, preoperatively and complications, 1-month postoperatively
   B. risky lifestyles and complications at 3 and 6-month follow-up
2. Efficacy of STRONG compared to TAU on health gain after 6 months and 2-year follow-up
3. Cost-effectiveness of the STRONG program on short and longer term (for 1. A+B and 2.)
4. Prevalence of SNAP factors in the potential study population and the potential for risk reduction.
5. Secondary analyses (Cohort analyses):
   A. Dose-response of SNAP factor improvement (number/type) and complications as well as predictors for complication-free hernia repair
   B. Dose-response of SNAP factor improvement (number/type) and health on long-term as well as predictors for improved health
   C. Associations between self-reported and validated lifestyles
   D. Associations between patient reflections of motivation, priority, self-efficacy, and lifestyle changes (Nested Qualitative analysis to the intervention group)

The hypotheses

The primary hypothesis is that STRONG halves the postoperative complications within 1 month compared to the daily routines.
Secondly, improvement of postoperative complications after 3 and 6 months, risky SNAP lifestyles, health, and the cost-effectiveness as well as improvement of health and costs after 2 years.
In addition, we hypothesise associations

- between preoperative patient reflections and successful change of SNAP factors
- as a dose-response of SNAP factor improvement and complications, as well as predictors for complication-free hernia repair
- as a dose-response of SNAP factor improvement and health gain, as well as predictors for a health gain
- between self-reported and validated SNAP factors.

The outcomes

The primary outcome is postoperative complications according to the Clavien-Dindo classification[4].
Secondary outcomes are improvement of risky SNAP lifestyles (self-reported and validated), risk evaluation (according to the lifestyle categories in the ASA-score[5]), patient reflections, health (measured by health-related quality of life, symptoms of frailty, and co-morbidity) and costs (measured as individual direct and indirect healthcare costs per patient based on perioperative costs, time back to work or reuptake of previous activities, total stay, and visits/contact to hospital and primary healthcare (from the national health registries).

The rationale

The clinical STRONG project is based on the rationale

- that risky SNAP factors increase postoperative complications
- that preoperative intensive mono-factor intervention prevents a part of the complications
- that successful SNAP factor intervention improves health on longer term

In real life, several patients have co-existing risky lifestyles. Hitherto, no RCTs have integrated all SNAP factors. Therefore, time has come to take a common grab of the five SNAP factors as in the STRONG prehabilitation and establish new knowledge of the effect on postoperative complications, health & costs, and patient reflections.

2b) The background (Reference numbers refer to the list in chapter 16)

Surgery remains the gold standard of treatment for many diseases including ventral herniation, but the outcomes are poor among patients with unhealthy lifestyles; thus, strategies to further optimise the perioperative care are increasingly important. The improved intraoperative techniques and enhanced recovery after surgery (ERAS), e.g., the ERAS protocol, are highly relevant[2] – including for ventral hernia repair[6]. However, there is a large untapped potential of risk reduction in the preoperative period, in particular the modifiable risk factors, which can be targeted with preventive strategies[7].

Systematic screening and related intervention for the SNAP factors prior to surgery are only fragmentally implemented in the surgical pathways[8]. This is despite the World Health Organization (WHO) recommendations and the surgical guidelines endorsing all patients to be offered evidence-based support for risk reduction, as despite the American Society for Anesthesiologists (ASA) inclusion of relevant details on alcohol, smoking and obesity in the international preoperative risk evaluation for adults, the ASA-score[5].

The SNAP factors each adds independently to the surgical risk with about 50% for daily smoking, alcohol > 2 drinks/day[9], and severe malnutrition[10] — similar to the risks related to severe cardiac, pulmonary, and kidney insufficiency. Obesity after both major and minor surgery[11,12] and low physical activity are followed by a 10–20% increase in complication rates[13]. Frailty is an example of co-existing SNAP factors with a major impact on the surgical outcome[14,15].

Pathophysiological mechanisms

Patients with an unhealthy lifestyle develop the same type of complications as all other patients, just more frequently[10,16,17,18,19,20]. The pathophysiology involves a comprehensive suppression of several organ systems of importance for surgical outcomes, such as low immune capacity, disturbed wound and tissue healing, cardiac and lung dysfunctions, and increased stress-response amongst others. These dysfunctions exist prior to surgery and add to the surgical trauma itself, often targeting the same organ functions. The increased postoperative complications are developed from the SNAP factors even without clinical diagnosis like alcohol-related liver disease and smoking-induced lung disease[9]. The organ damage is often subclinical and takes place at the cellular level, thus reducing the extra capacity that usually supports the patient successfully during surgery and recovery.

Need for a combined programme

The risk at surgery increases with co-existing risk factors, and thereby even minor surgery can lead to a bad outcome, but no published integrated program exists targeting all five SNAP factors[13,21] except for the STRONG program[22].

We and other research groups have previously shown that an intensive intervention aiming at complete smoking or alcohol abstinence for 4 to 8 weeks halves the postoperative complication rate and indicates a sustained effect for longer time[23,24]. For weight reducing programs, again the intensive intervention has a better effect on preoperative obesity than shorter programs[25].

Preoperative inspiratory exercise for 1–2 weeks reduces postoperative pneumonia[26], while malnutrition intervention before surgery is part of the international guidelines[10,27]. Physical exercise improves respiratory fitness and functionality and followingly reduces postoperative recovery, but the impact on complications has been disappointing[28,29,30]. However, a recent systematic review has identified a reduction of lung complications after major lung and cardiac surgery, but it is still inconclusive for other surgical interventions[31]. The four randomised trials on frailty intervention (exercise and nutrition) show no effect on postoperative outcomes[32,33,34,35].

Smith and co-workers identified no randomised trials evaluating the efficacy of preoperative obesity intervention outside bariatric surgery[36]. After their literature search period, one randomised trial has been published. Despite a significant preoperative weight reduction in the intervention group, the development of postoperative complications was similar in both groups[37].

Overall, there is a call for prehabilitation including more SNAP strategies, and the European Hernia Society strongly recommends conduction of high-quality studies of prehabilitation in relation to ventral hernia repair [38].

As the first worldwide, our research group tested a combined intensive smoking and alcohol cessation intervention, which was well received by the patients[39]. The intervention took place shortly prior to the cancer surgery and thereby probably reduced the overall expected effect on postoperative complications (64% versus 70%), despite the high successful quit rates (51% versus 27%). Interestingly, the intervention group had lower number of patients with three or more complications on short and longer term[40].

We have also identified a minimal impact only of the social gradient on the effect of the national intensive smoking cessation intervention[41]; thus, far from the social gradient among smokers - and that program is used in the STRONG project. In addition, the social inequality in health promotion success could be minimised through positive/selective involving procedures and support during the total intervention. A full SNAP intervention like the STRONG program is further supported by the results from secondary analyses that intensive mono-factor intervention on smoking and alcohol did not affect other risky lifestyles among surgical patients[42].

We are conducting a systematic review with a network meta-analysis on published randomised trials evaluating the effect of preoperative lifestyle intervention for two or more co-existing SNAP factors (PROSPERO: CRD42022282611). After screening the almost 18,000 eligible studies, the result of 19 trials is disappointing regarding the number of SNAP factors intervened. There is not published randomised trials aiming at all 5 SNAP factors but there is one study on smoking + alcohol cessation intervention from our group[39], one on smoking + respiratory training[43], and the rest on physical/respiratory training + any nutrition intervention. We experience a general lack of predefined inclusion criteria on nutritional level and physical activity. The risky SNAP factors are carefully predefined in the STRONG project.

Cost-effectiveness

It is important to consider the costs of the STRONG in relation to the surgical outcomes. The cost-analyses are conducted via the national health registries including the individual DRG registry together with data collected in the project.

State of the art for Ventral hernia repair and SNAP factors

Internationally and nationally, more and more scientific activities are performed aiming towards evidence-based guidelines, including uniform reporting of complications and patient reported outcomes as well as the use hereof[44,45]. The Danish Hernia Database plays an important role in implementation and follow-up of these activities[46]. It does, however, not include the SNAP factors, yet.

A major systematic review on ventral hernia repair has recently updated the evidence, including for the SNAP factors. Two randomised trials were identified for smoking, one Danish study by Sørensen and co-workers reporting no effect on would complications after only low intensive interventions: Simple advice versus simple advice with reminding (4/48 = 8% and 6/101 = 6%)[47]. The other trial is Swedish by Lindström and co-workers[48]. They reported any complication after ventral hernia repair which, however, constitutes a sub-group on 38 patients, 21 in the intervention group receiving intensive intervention and 17 in the control group receiving usual care, respectively. The total complication rate after hernia repair was halved after smoking cessation intervention; 6/21 in the intervention group versus 10/17 in the control group or 29% versus 59%. The complications were mainly wound complications (Authors personal information). No randomised trials were identified for preoperative obesity intervention outside bariatric surgery for nutrition or physical activity as mono-factor intervention. Alcohol intervention is not mentioned at all. Nevertheless, one trial used combined intervention aiming at both obesity and low fitness[49], followed by a 2-year follow-up[50] (see next paragraph).

More than twenty years ago, the question of a higher impact of co-existing SNAP factors on the complication rate than the individual factors alone, was evaluated in a cohort of 26,961 adult patients receiving hernia repair[51] . They exclusively evaluated perioperative respiratory events and found an incidence of 3.1% and a risk rate of 2.4 (95% CI: 1.8-3.2). These outcomes were even higher among overweight smokers below the age of 40 years; 8.0% and a risk rate of 6.3 (4.3-9.2). For comparison, the incidence of respiratory events was only 1.3% among those without smoking and overweight. Another recent cohort study with 55,240 patients evaluated the surgical site infection rate after hernia repair[52]. Here, they found a dose-response with increasing rates at increasing BMI cut-of levels among smokers. BMI (OR 1.04; 95% CI 1.03–1.04; $p < 0.001$) and smoking (1.51; 1.37–1.67; $p < 0.001$) were the two most modifiable risk factors independently associated with this complication. Interestingly, the incidence was only 2% among non-smokers with BMI < 24.2, and 12% for smokers with BMI > 42.3. It would be clinically relevant to evaluate if an 'opposite' dose-response could be identified also during intervention for SNAP factors by the STRONG program. This objective is included in the present STRONG project.

Risky lifestyle plays a further role for recurrence of ventral hernia[53]. A recent systematic review identified smoking (OR 1.34; 1.03-1.74) and BMI above 25 (2.51; 1.37-4.60) as preventable predictors for hernia recurrence as well as complications at the primary repair[54]. Reduction of postoperative complications via successful prehabilitation would further add to the potential for recurrence reduction. However, alcohol and malnutrition were not included in those analyses.

Vi have identified only one study on co-existing SNAP factors performed by Liang and co-workers, who have reported a trend towards less wound complications of an up to six months program with physical exercise + weight reducing intervention: 7% versus 18%, p=0.1[49]. At 2-year follow-up, there was no effect regarding complication- and recurrence-free patients, 73% versus 66%, p=0.4[50].

2c) STRONG-Hernia is powered for evaluation for postoperative complications

A prior step towards the present STRONG-Hernia project has been taken by the ongoing STRONG – Cancer project, in which we are evaluating the effect of the STRONG program on lifestyle improvement and related risk reduction, exclusively from improvement of SNAP factors. STRONG-Cancer is an RCT recruiting a smaller group of patients scheduled for urothelial cancer surgery after preoperative neoadjuvant therapy. In a nested interview study, we collect the preferences among the recruited patients, their relatives, and staff (www.clintrials.gov NCT04088968; the Danish Scientific Ethical Committee H-20081571; the Danish Data Protection Agency P-2020-95)[55]. That trial does not aim at and therefore is not powered for evaluation on postoperative complications, health, and cost-effectiveness, like the present STRONG-Hernia project.

However, the positive process is useful for the present project on ventral hernia repair. Thus, the present project on ventral hernia repair is not a repetition of another project but adds completely new and clinically important knowledge to the research area of prehabilitation regarding SNAP factors.

3. Methods

3a. Designs, framework, analyses, randomisation, and control group

Design

The project uses multiple designs to test the hypotheses and answer the research questions presented in Chapter 2a, including a randomised design with nested qualitative, costs, and secondary cohort analyses, see figure 1.

Figure 1. Trial profile for STRONG-Hernia; CG= Control group, EXCL: Exclusion, FU: Follow-up, IG: Intervention group, OP: Operation, R: Randomisation, SNAP: Smoking, Nutrition (malnutrition and/or obesity), Alcohol, Physical activity.

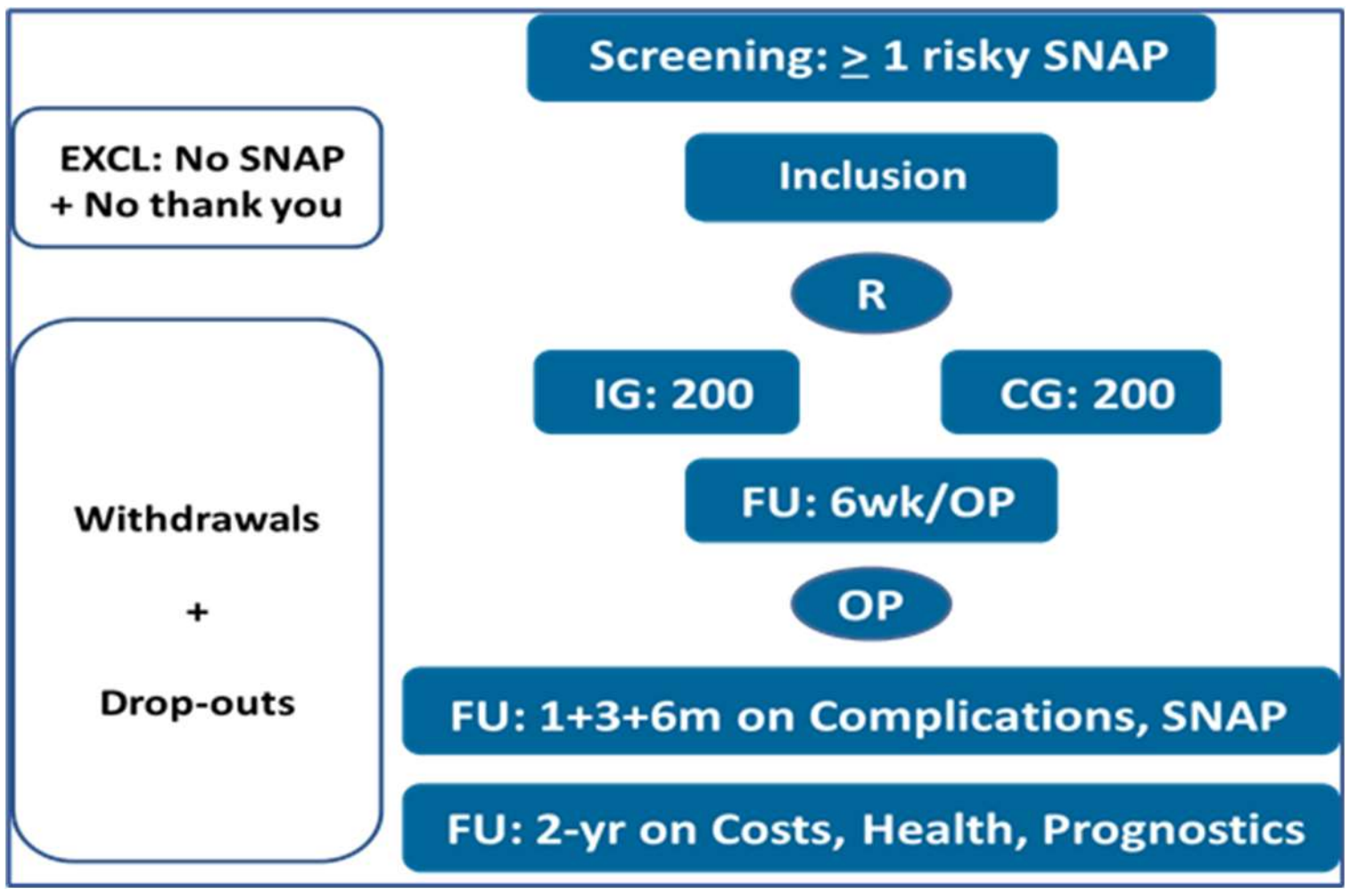

Theoretical framework

Intervention targeting reduction of risk for postoperative complications is closely related to the preoperative recovery of dysfunctional organ systems prior to surgery, see figure 2.

Figure 2. Framework: Functionality and organ functions in the perioperative period. (OP: Operation. TAU: Treatment as usual)

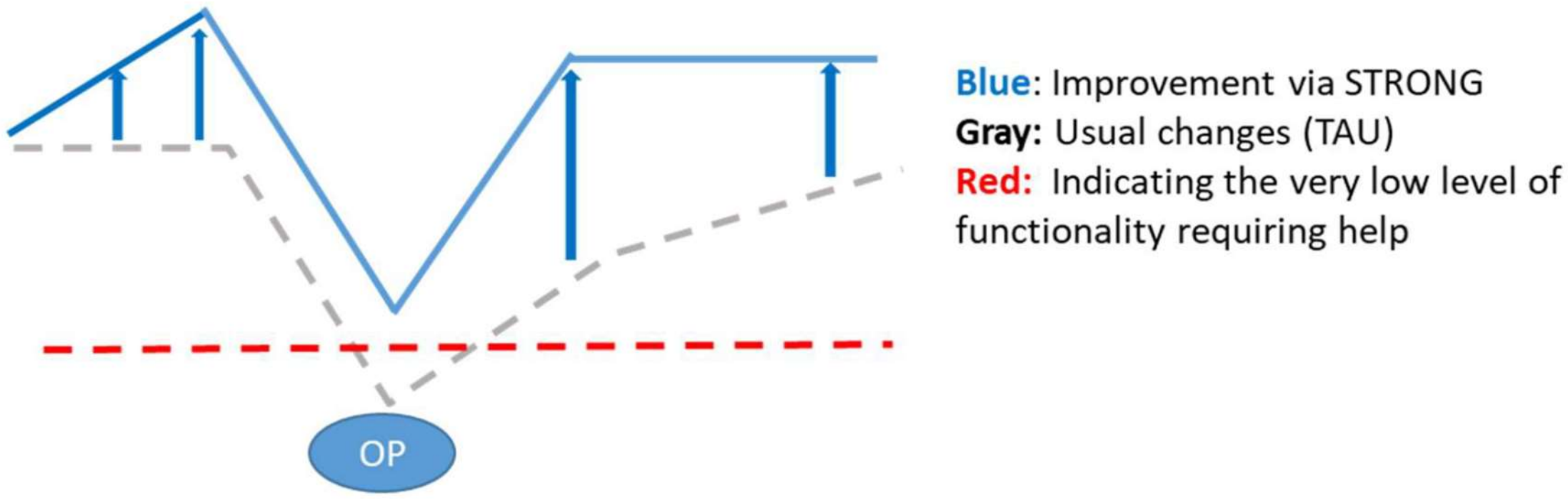

Our intensive SNAP intervention is tailored to meet the individual patient's need for risk reduction at surgery via the intensive 6-week STRONG program including from one to five co-existing risky SNAP factors per session[22]. The patients in the intervention group is introduced to the STRONG program as previously described in the World Health Organization publication 'Engage in the process of change'[56] and elswhere[22,40].

Analyses
Analyses are done blinded as Intention-to-Treat (ITT). We will use Fisher's exact test for frequencies, Mann-Whitney for continuant variables, and $p < 0.05$ is chosen for statistical significance. To benefit future meta-analyses, we will also give the parametric results. This would also meet the tradition that costs are usually reported with means and standard error (SE), mean difference (MD), and 95% confidence interval (CI). Bootstrapping procedures are used to calculate the cost-effectiveness plane and the acceptability curve by repeatedly resampling the data (1,000 incremental cost and effect pairs). Sensitivity analyses will be performed for differences in local standards for the control groups among the surgical centres. Analyses as per-protocol, dose-response, prediction, sensitivity, and the other secondary analyses are done by logistic regression models including control for confounders and effect-modifiers and reported as Risk Rates with 95% Confidence Interval (CI). The results are considered significant if the CI does not involve 1.00.
Absolute (ARR) is calculated as the events in the control group minus the events in the intervention group, and the relative risk reduction as the ARR divided by the events in the control group. The number needed to treat was calculated by 1/ARR. We will present the mortality as Kaplan-Meier plots and test by the log rank test.
In case of dropouts, the primary outcome is collected from the medical record and a telephone follow-up after acceptance from the patient. In case of missing health data, the lifestyle at baseline will be imputed as the results in order not to over-estimate the results of changing lifestyle. This would expectantly impact the results to a similar degree in both groups.
All quantitative analyses are performed in SPSS Statistics® version 22.

The qualitative analyses are done using Kirsti Malterud's approach of systematic text condensation[57]. After import into NVivo® qualitative data analysis software version 11, the systematic text condensation in the four steps takes place: 1) Total impression of all answers and identifying preliminary themes, 2) coding by identifying and sorting meaning units, 3) condensation into code groups, and 4) synthesising the condensates into a story grounded in the empirical data.

Randomisation
The randomisation to the intensive STRONG Program or standard care takes place via the Redcap program as recommended in the Capital Region with stratification for each centre as well as for one, two or three-to-five co-existing risky SNAP factors and computer-generated blocks ranging from 2 to 5. The randomisation system is accessible around the clock via computer.

The control group
The control group receives the standard care regarding the SNAP factors. This includes the participant information, weight loss and smoking cessation initiatives, according to local routines, and access to support offered outside the project.
The project does not involve placebo intervention in the control group.

The practical procedures, studies, and outcomes
The 400 patients are recruited at the involved surgical departments:

- Herlev Hospital (Jacob Rosenberg),
- Regionshospitalet Horsens (Hans Friis-Andersen),
- Sjællands Universitetshospital Køge (Frederik Helgstrand),
- Sjællands Regionshospital Holbæk (Birgitte Brandstrup).
- - more departments – also in the private sector – may be included if necessary and after extended approval from the Ethical Scientific Committee and the Danish Data Protection Agency.

Standard protocol for SNAP at Horsens: Weight loss information and smoking cessation as BMI < 30 and smoking cessation (without verification by urine cotinine test) are mandatory.

Standard protocol for SNAP at Zealand University Hospital, Køge: Weight loss information, smoking cessation - active surveillance, and follow-up by nurses aiming at BMI < 35 and smoking cessation (without verification by urine cotinine test) mandatory.

Standard protocol Herlev University Hospital: The aim is a general optimization prior to surgery, including regarding BMI and smoking (without verification by urine cotinine test).

Standard protocol Zealand Regional Hospital, Holbæk: The aim is a BMI under 35 and optimization of any comorbidity prior to surgery. For operation of major hernia of 10 cm or more, it is recommended to stop smoking six weeks preoperatively (without verification by urine cotinine test).

After informed consent, patients are included and allocated to either the 6-week STRONG intervention or the control group. Data collection takes place at baseline, after 6 weeks/at time of surgery, and postoperatively after 1, 3 and 6 months at the local surgical department. The data are included in the databank in Redcap, and blood and urine samples are transferred to the established project biobank at the Parker Institute. For the long-term follow-up on health, further data are drawn from national health registries up to 2-years after the inclusion (project flow in figure 1).

The studies

The project is constituted by several sub-studies all based on data from the RCT with 400 patients as specified in Chapter 2.

Outcomes and data collection (Table 1)

<u>Postoperative complications (within 1, 3, 6 months after surgery):</u> The primary outcome is the number of patients with > 1 complication, defined by requiring documented treatment, categorized in accordance with the Clavien-Dindo grading[4] and the Comprehensive Complication Index (CCI)[58]. They include adverse events and side effects. Secondly, length of stay, second surgery, revisits and – admissions at hospital, visits in primary care, time back to work or previous activities.

<u>Successful risk reduction at follow-up/at surgery:</u> Defined as SNAP below risk level validated as described in chapter 2; for obesity BMI < 30 or at least 5% and max 10% loss of body fat mass preoperatively (0.5–1 kg/weekly without developing malnutrition). Data are collected via logbook and validation.

For frailty: In addition to pl-protein[59], the frailty level (0-5, 0 is best) is measured by Fried's Modified Frailty Score according to Blammers[15] as that is associated with development of complications[60,61]. It includes five criteria:

- Slowness: Performance time in the walking test,
- Weakness: Hand grip strength measured by a dynamometer,
- Exhaustion: Responding "All the time" or "Often" to the item "I feel everything is an effort / I can't get going" in the Hospital Anxiety & Depression Scale[62].
- Weight loss: Self-reported 5 kg over the last year
- Low activity: Below 3 MET

<u>Health (at baseline, after 6 weeks/at surgery, and 6 months postoperatively):</u> This is measured by the health-related quality of life measured by EQ-5D[63] with 5 dimensions and the overall health measured with a scale from 0 to 100 with a higher score representing a better health. Within 2 years of follow-up, the morbidity is measured as visits and stay at hospital as well as visits in primary care and prescribed medicine and grouped diagnoses groups as well as mortality.

Prevalence of SNAP factors at baseline and the potential for risk reduction. Data are collected from the medical records. .

Costs (after 1 month, 6 months, and 2 years): Data on direct healthcare costs are collected for each individual patient from the National Hospital Costs Registry (DRG-registry) and the National Registry on Primary Healthcare. Data on cost-effectiveness includes the EQ-5D[63] on Health outcome as described above.

Qualitative outcomes for the intervention group: The patient reflections and expectations regarding motivation, priority and self-efficacy for lifestyle changes are collected prior to the intervention. The information is measured at a scale from 0-100 (100 being the highest level). Free text is collected regarding the benefits and challenges of continuing and quitting the lifestyle.

Table 1: Data collection for all included participants at baseline, after 6 weeks/surgery, and at follow-up 1, 3 and 6 months postoperatively, as well as after 2 years.

Measurements	Inclusion	Week 6 / at surgery	Follow-up 30 days	Follow-up 3+6 months
**Socio-Demographic data (patient reported)**				
Age and education (years), sex, housing, living alone, working conditions, level of daily activities	X			
**SNAP Screening for risk: yes/no (patient reported)**				
Smoking, Nutrition, Alcohol, Physical activity	X			
**Status (patient reported)**				
Smoking (nº cigarettes/day, Nutrition (intake), Alcohol (nº drinks/week), Physical activity (min/day)	X	X	X	X
EQ-5D (HRQoL)	X	X	X	X
Adverse events		X	X	X
**Status (validated)**				
Blood + urine: Alcohol & Nicotine biomarkers, Hgb, Albumin, Protein, ASAT, Bilirubin	X	X	X	X
Weight and bio-impedance, height,	X	X	X	X
Frailty score (weight loss, handgrip, walking test, exhaustion, level of activity)[60,61]	X	X	X	X
ASA-score	X	X	X	X
**Postoperative complications**				
Complications /yes/no)			X	X
Clavien-Dindo grade and CCI			X	X
Stay in hospital			X	X
Second surgery			X	X
Revisits and readmissions at hospital			X	X
Visits in primary care			X	X
**Long-term register data on Health and Costs**			Up to 2 years	
Morbidity (National patient registry)			X	
Mortality (Death certificate registry)			X	
Individual DRG costs at hospital (DRG registry)			X	
Primary care usage (Primary care registry			X	
Prescribed medicine (Medicine registry)			X	

3c. Interventions

Intervention group: Patients in the intervention group receive minimum five educational sessions over 6 weeks (i.e., about weekly) as an integrated prehabilitation program tailored to meet the

individual patient's need for risk reduction at surgery, see Table 2 and 3. It is introduced via the surgical 'Engage in the process of change'[55]. The smoking intervention follows the Gold Standard Programme GSP[64], which has been translated to the standardized alcohol cessation intervention[39,65], exercise training programme, and nutritional intervention[66].
Project nurses from the participating centre, who have taken part in a 5-day educational program followed by practical training in the Gold Standard Programme (GSP), provide the intervention. The project leader (SVL) of the trial ensures that counsellor follows the principles of the GSP program by regular discussion of the intervention.

Table 2. Weekly sessions after inclusion (Preop: preoperatively)

	Week 1	Week 2	Week 3	Week 4	Week 5	Week 6 Follow-up + continued preop.
**Smoking cessation**	X	X	X	X	X	X
**Nutrition program (malnutrition)**	X	X	X	X	X	X
**Nutrition program (obesity)**	X	X	X	X	X	X
**Alcohol cessation**	X	X	X	X	X	X
**Physical Activity**	X	X	X	X	X	X

Table 3. The patient education program in the Gold Standard Programme (GSP)

Week number	Education subject
**1 (at inclusion)**	**Level of motivation, ambivalence, pros and cons (see Appendix)**
**2**	**Dependence, withdrawal symptoms (experience and expectations)**
**3**	**Relapse (description and management)**
**4**	**Benefits of short- and long-term lifestyle change**
**5**	**Continued change of lifestyles (handling risk of relapse)**
**6 and follow-up**	**Continued education based on the current conditions**
**+ continued preoperatively**	**Continued education based on the current conditions**

At each meeting we look into: The status and follow-up on the 'homework' via logbooks as well as challenges and successes. Patients are asked if they have experienced side effects of the pharmacological support. Potentially unknown side effects are reported and, if serious, may lead to early termination of the trial.

The specific physical activity includes 5 minutes respiratory exercise and 25 minutes activities chosen by the patient from a menu, thus adding up to 30 minutes per day. It starts on the first meeting and the patient walks through the complete 30 minutes program supported by the counsellor.
The specific nutrition program is adjusted to the patients individual need ensuring the complete intake required to change lifestyle in accordance with the individual needs.

The pharmaceutical support for smoking cessation intervention follows the national recommendations that have successfully been used in Danish studies of surgical patients[40,67]. It is emphasized that the entire program is adapted to the individual patients - including guidance on supportive medicine - and that all pharmaceutical support is an offer. It includes personalised Nicotine Replacement Therapy (NRT) in accordance with the internationally acknowledged Fagerström test for nicotine dependency[68] and patient preferences. Pharmaceutical support for risky drinkers also follows the national recommendations, successfully been used in Danish studies of surgical patients[40,60]. It includes thiamine (300 mg daily) and combined B vitamins, alcohol withdrawal prophylaxis and treatment (chlordiazepoxide 10 mg as required in national guidelines) and low dose of disulfiram (200 mg × 2 weekly) supervised at weekly meetings (administered only if the patient has a negative alcohol breath test). The study medication is provided for free. The intervention group is followed during the intervention by additional measurements, Table 4.

Table 4: Additional measurements in the intervention group related to the SNAP factors of the individual participants (AUD: Alcohol use disorder; AWS: Alcohol withdrawal symptoms; CIWA-aR: Clinical Institute Withdrawal Assessment for alcohol-revised; CO: carbon-monoxide; Diagnostic NRT: nicotine replacement therapy; TLFB: timeline-follow-back)

Measurements (Intervention group, exclusively)	Week1	Week 2	Week 3	Week 4	Meet 5	Week 6⁺
Motivation and reflections	X					
Adverse events	X	X	X	X	X	X
Smoking						
Cigarettes per day (Nº)	X	X	X	X	X	X
Tobacco dependence (Fagerström criteria)	X					
Use of NRT (mg)/other craving reducing medicine	X	X	X	X	X	X
Validation by CO concentration (breath)	X	X	X	X	X	X
Nicotine biomarkers in urine	X		X		X	X
Alcohol						
Alcohol per week (units 12 g, TLFB)	X	X	X	X	X	X
Alcohol dependency (AUD criteria)	X					
Alcohol concentration (breath test)	X	X	X	X	X	X
Alcohol biomarkers (blood + urine)	X		X		X	X
If withdrawal symptoms (CIWA-aR)	X	X	X	X	X	X
Use of AWS prophylaxis (mg)	X	X	X	X	X	X
Nutrition						
Food intake according to plan	X	X	X	X	X	X
Weight and bio-impedance	X	X	X	X	X	X
Validation by Nutrition profile (blood)	X		X		X	X
Physical Activity						
Physical activity according to plan	X	X	X	X	X	X
Steps walked per day	X	X	X	X	X	X
Walking test	X		X		X	X
Hand grip strength	X		X		X	X

<u>Control group:</u> All patients in the control group receive the project information about the study. They receive the routines of the involved departments (e.g., smokers may receive the Very Brief Advise and referral to a municipal clinic for smoking cessation intervention). All patients are free to use any support offered outside the project, including the free access to lifestyle intervention in their municipality.

<u>All participants:</u> All meetings are planned in connection with scheduled visits in the outpatient clinic or at admission to hospital when possible and in other cases the transportation for the weekly meetings will be reimbursed. All participants have telephone access to the research nurse during the project.

All receive routine procedures on general patient information, thromboembolic prophylaxis and antibiotics, anaesthesia, surgical intervention, and postoperative care as used at the centre. All patients are evaluated at inclusion and 6 weeks after/at surgery as well as 1, 3 and 6 months postoperatively, Table 1.

To find out if eligible patients who decline to participate in the trial differ from those enrolled, we ask for permission to follow them perioperatively via the medical records and for 2 years in the health registries. Those who accept are asked to provide informed consent.

4. Statistical considerations: Power calculation

The power calculation is based on the literature as presented above, thus aiming at halving of postoperative complications after intensive programs for smoking, alcohol, and malnutrition. The impact of physical activity is lower, and obesity is sparsely investigated but targeting co-existing SNAP like STRONG should increase the effect due to interactions. Therefore, the main hypothesis is clinically relevant. For ventral hernia in high-risk patients, STRONG would expectantly half the overall complication rate from conservatively 20% to 10%[47,48,49,51,52]. When using 80% power and 2 x alpha=0.05 we get 2 x 199 participants. This also covers the secondary outcomes regarding lifestyle improvements and costs.
In case of dropping out, the patients are asked to allow follow-up via the medical record system and a telephone interview. It is our experience from previous studies that 90% the drop-out patients accept this. Therefore, the development of complications as the primary outcome will be available for close to all randomised patients. We use block randomisation in rather small groups, which would only to a minimal degree impact the distribution between the intervention and control groups.
In study 4 the prevalence of risky SNAP factors on all patients that potentially could be screened for eligibility in study 1 (about 2 x the included numbers = 800 patients) will be reported as frequencies and 95% CI and analyzed for the potential maximum risk reduction and effects, if they would have received a related STRONG intervention. If possible, the potential will be analyzed for the most frequent combined risk factors.

5. Participants and criteria

Inclusion criteria: Consecutive adult patients scheduled for ventral hernia repair (umbilical, incisional and epigastric hernia) and screened positive for at least 1 risky SNAP factor at least 4 weeks prior to the date of surgery are eligible for participation.

Exclusion criteria: Other ventral hernias, (para-stomal hernias as well as giant ventral hernias with defect > 8cm), pregnancy and breastfeeding, allergy/contraindications to pharmaceutical and nutritional support and exercise in STRONG, not able to give informed consent (e.g., age<18 years, severe mental illness, compromised consciousness, and language challenges), previous alcohol delirium/seizures and withdrawal of consent.

6. Risks, side-effects, and inconvenience

6a) Considerations

Participation has only few risks such as side-effects of the well-established pharmaceutical support in the intervention group. The medicine administration follows the national guidelines, including the established indications for use. Side-effects are reported to the Health Authorities.
Collection and storage of sensitive data for patients may be followed by a risk of data leak. This risk is lowered by carefully following the Danish guidelines for each procedure. The study will be approved by the Danish Data Protection Agency, and data will be stored in the Redcap (as recommended by the Region) on secured and logged servers according to Danish regulations.

In addition, for the intervention group changing of lifestyle could be considered uncomfortable for some period, however, important efforts are taken to reduce this by information, patient education, motivational support and in case of quitting high alcohol intake and daily smoking also prophylaxis against possible withdrawal symptoms.

However, this seems to be balanced by the possible effect on the reduced risk at surgery on short term and a possible effect on health on longer term. Furthermore, the participants have access to 24 hours/7 days a week open hotline (e-mail or phone) to the clinical project staff.

All participants are free to use other healthcare services to support lifestyle changes during the study period if wanted. The patients are asked about this at the follow-ups.

There might be risks or side-effects not previously known. Therefore, we ask the participants to inform about experiences of health problems during the study period. If we identify new types of side effects, the participant will be informed in order to consider the continuation of participation.

6b) Discontinuation

Participant withdrawal: A participant may withdraw from the study at any time without this impacting on any future investigations and/or treatments at the site.

Individual participant discontinuation: The investigator may discontinue any participant's participation for any reason, including an adverse event, safety concerns or failure to comply with the protocol.

Discontinuation of entire study: The principal investigator has the right to terminate this study at any time. Reasons may include the following, but are not restricted to unsatisfactory fulfillment the design, participant enrolment, keeping the timeschedule, or agreement with the description in the application.

7. Sampling of blood and urine in our research biobank for biomarkers

All participants have blood and urine tests sampled at baseline and the four scheduled follow-ups for the identification of alcohol- and tobacco biomarkers (B-PetH, U-EtG and U-cotinine), for routine nutrition profile analyses (Pl-Protein and Pl-Albumin), and routine analyses on B-Hemoglobin, Pl-ASAT, Pl-Bilirubin as well as for measurement of possible new markers developed during the project period. In total 30 ml blood and 10 ml urine are sampled at each meeting (inclusion and the 4-5 follow-ups with 1-3 months between). Half of the samples is kept for the analyses of new biomarkers possible developed during the project period (see below).

The routine analyses are performed at the local centres. The analyses of the established biomarkers on tobacco and alcohol are performed together for one factor at a time, mainly after collection of all samples. Therefore, it is necessary to collect and store the samples (separated into 1.0 ml in 1.8 ml cryotubes at minus 80° C placed at Nordre Fasanvej 57, vej 8, indgang 2, kælderen, Rum: nr: 60.0.11 Bispebjerg-Frederiksberg Hospital) during the project period until analysed. The bio-marker analyses are expected to be performed at the Parker Lab, same address. Left-over samples in the individual tubes used for routine and marker analyses are destroyed immediately afterwards.

As mentioned above, new markers may be developed during the project period, and we would therefore keep half of the samples (i.e., 5x1 ml whole blood and 2x1 ml EDTA plasma, 2x1 ml citrate plasma, 2x1 ml serum, and 5x1 ml urine) stored for further analyses related to measurement of markers for risk reduction at surgery during the study period. Left-over samples will be destroyed after end of the study period (31.12.2033).

In addition, the participants are separately asked about giving informed consent to keep the possible left-over samples stored in a completely anonymised form for future and not yet specified research. It is understood that the samples can be used in future research only after approval from the Scientific Ethical Committee and the Danish Data Protection Agency. If the participants do not give

the separate informed consent, all biological material will be destroyed at the end of the project (as described above)

8. Collection of information from the medical records

Prior to informed consent (but after patient contact in relation to the present hernia pathway with the involved surgical clinics), the medical records are screened for diagnoses and SNAP factors. This information is used by the research group to identity possible participants to be informed about the study and for study 4. After informed consent, the patient characteristics of age, gender, diagnosis, planned treatment including surgery, co-morbidity, risk factors and present lifestyle profile as well as the descriptions of surgery and anaesthesia, the stay in hospital, possible complications and related intervention, conditions, re-admissions, visits in the out-patient clinic, and other similar activities in the perioperative period are collected. The consent gives the responsible researchers, the sponsor and representatives, and eventual supervisory authority direct access to obtain information in the patient's medical record, etc., (including electronic medical records), in order to see information about the participant's health, which is necessary as part of the implementation of the research project and for control purposes, including self-control, quality control and monitoring, which they are obliged to carry out.

9. Data Handling

9a) Handling and storage

During the collection period, the data are stored at the secured Redcap in the Region H, as recommended[69]. Data analyses are performed blinded and there will be no personal identification in presentation of results. After finalizing the project in 2033, all person identification including the project number will be deleted, so only the anonymous results will be kept for documentation. The study will before start be approved by:

- The Scientific Ethical Committee in Denmark.
- The Danish Data Protection Agency. The study follows the General Data Protection Regulation (GDPR, Databeskyttelsesforordningen) as well as the Data Protection Act (Databeskyttelsesloven). Data Processor Agreements are signed by the partners participating in data collection and bio-analyses.
- The protocol is registered at the www.clinicaltrials.gov.

9b) Organization

The study is organized with local STRONG teams for Hernia repair at the surgical departments, the Health Economy team at University of Southern Denmark, the patient panel and the international Advisory Board as well as the Qualitative team and the national STRONG Coordination at the WHO-CC, Parker Institute, Bispebjerg-Frederiksberg Hospital. The analyses, biobank and project administration take place at the Parker Institute.

Detailed information of the partners is given here:
The Hernia Repair team under leadership of Professor Jacob Rosenberg:

- The Department of Surgical Gastroenterology at Herlev Hospital. In addition to Professor Jacob Rosenberg, this team includes postdoc Siv Fonnest and the young researcher Sofie Skovbo Jensen (PhD-student from July 2023) and a halftime trained project nurse.
- The Hernia Center at Horsens Regional Hospital with the local Project Leader Associated Professor Hans Friis-Andersen and a halftime trained project nurse.
- The Department of Surgery, Zealand University Hospital in Køge with the local Project Leader Frederik Helgstrand, Clinical Lecturer and Associated Professor and a halftime trained project nurse.

- The Department of Surgery, Zealand Hospital in Holbæk with the local Project Leader Associated Professor Birgitte Brandstrup and a halftime trained project nurse.

The Qualitative team of Senior Researcher Susanne Vahr Lauridsen and Anthropologist Rie Raffing at WHO-CC, The Parker Institute Bispebjerg-Frederiksberg Hospital.

The Health Economy team under leadership of Professor Kim Rose Olsen at The Danish Centre for Health Economics, University of Southern Denmark

The national STRONG Coordination under leadership of Professor Hanne Tønnesen, Principal Investigator, Senior Researcher Susanne Vahr Lauridsen (STRONG intervention coordination), Professor Berith Lilienthal Heitmann (public health epidemiologist and data- and biobank manager); biostatistician, and project secretariat, administration (Claus Bomhoff) at the WHO-CC, The Parker Institute Bispebjerg-Frederiksberg Hospital.

Further partners are the established STRONG Patient Panel that meets 2-4 times a year and the Advisory Board of international clinical researchers in prehabilitation and representatives from patient organization and WHO, meeting twice a year

10. Economy

This is an investigator initiated clinical study.
The principal investigator Hanne Tønnesen has received 5.0 million DKK from the Insurance Company Danmark for salary og project staff, nutritional and pharmaceutical support, and lab analyses. The grant is administered by the Bispebjerg-Frederiksberg Hospital, Region H.
Furthermore, the Novo Nordic Foundation has supported the project with a grant of 0.6 million DKK.
The project staff has no relation to the grant-giving organization, which has no impact on the project.
The co-financing from the Parker Institute at Bispebjerg-Frederiksberg Hospital covers office facilities and administration as well as bio-banking.

11. Payments for participation

There is no payment for participation.

12. Recruitment of participants and informed consent

In connection with the initial planning of surgical treatment in the outpatient clinics, the project nurses will screen patients referring to the inclusion and exclusion criteria. For patients meeting the inclusion criteria and none of the exclusion criteria, a project nurse will approach the patient and offer more information about the project. If they are interested in hearing more about the project, they are contacted by the local project team. Before this meeting, the patient is informed that family members and/or other important persons are welcomed on invitation of the patient. Interested patients will have oral and written information about the study and an invitation to participate. The patient information follows the guidelines from the National Committee on Health Research Ethics.

During the meeting, the oral and written participant information is given by the project members, and there is time for questions and answers. The dialogue takes place in a private and neutral room close to the out-patient clinic. Relatives or another relevant person (‘bisidder’) are invited to participate – depending on the patients’ wishes. The patients take the time necessary for consideration in the hospital or at home, normally up to a few days.

All participants are included after informed consent and can withdraw their consent without further explanation and without influence on the treatment and care in the department. Patients, who withdraw the informed consent or say no thank you to participate in the randomised trial are asked about a separate informed consent for being followed-up via the medical records and the health registries.

The informed consent is collected timely to allow for 4-6 weeks prehabilitation with the STRONG program. If the operation is postponed, the prehabilitation period is extended until surgery (e.g., 8-

	2023	2024	2025	2026
Protocol article writing	X			
**Study 1-A** Inclusion & data collection	X	X	X	
Analysing +Article writing	X	X		
**Study 1-B** Data collection	X	X	X	X
Analysing + Article writing		X	X	X
**Study 2** Data collection		X	X	X
Analysing + Article writing			X	X
**Study 3** Data collection		X	X	X
Analysing + Article writing				X
**Study 4-A** Analysing + Article writing			X	
**Study 4-B** Analysing + Article writing				X
**Study 4-C** Analysing + Article writing		X		
**Study 4-D** Analysing + Article writing		X		
Running information of the project	X	X	X	X

12 weeks). They are also welcomed to use the project hotline in case of further questions and considerations. Participants are included only after informed consent and would then be randomized to either the intervention or control group.

13. Dissemination of results

All results will be published – positive, negative, or inconclusive, including in www.clinicaltrials.gov. The results will be disseminated for each sub-study after its finalisation. Authorships follow the Vancouver Criteria. Furthermore, the results will be spread to the public at the website, by lectures and through the participant's networks. The timeline is depicted in table 5.

Table 5. Overview of the timeline for the STRONG-Hernia project.

14 Scientific Ethical Considerations

The risk of participation is very low or negligible, and we anticipate that the intervention group will benefit from participation. The control group will receive standard care. The inconvenience that sometimes follows a change of lifestyle is often short and efforts are taken for prevention.
This project aims at halving the complication rate after surgery on short term and improve the health on longer term for future patients. However, patients in the intervention group may benefit from these possible effects already during the study period. All patients may feel positive by the extra examinations and measurements as well as the open access to the project staff during the project period. Overall, the low risk of participation seems to be well balanced by the benefits, thereby justifying running the project.

The project is covered by the usual patient insurance for all patients in public as well as for the possible future centres at private hospitals.

15. Appendix: Data collection forms as integrated in the Redcap (in Danish)

16. Reference list

[1] Wouters D, Cavallaro G, Jensen KK, East B, Jíšová B, Jorgensen LN, López-Cano M, Rodrigues-Gonçalves V, Stabilini C, Berrevoet F. The European Hernia Society Prehabilitation Project: *A Systematic Review of Intra-Operative Prevention Strategies for Surgical Site Occurrences in Ventral Hernia Surgery.* Front Surg. 2022;9:847279.
[2] Kehlet H. *Enhanced postoperative recovery: good from afar, but far from good?* Anaesthesia 2020; 75: e54-e61
[3] Tønnesen H Raffing R, Svane JK, Lauritzen JB, Thind PO, Lauridsen SV, Wernerman SO, Fagerlund MJ, Wiksell R, Berman AH, Combalis A, Lozano L, Fernández Valencia JA, Santiñà M, Adami J, Spies CD. *Clinical Evidence and Knowledge Syntheses: Lifestyle intervention in the perioperative process through digital service - Live Incite.* EU Report 2017, Horizon 2020-727558.
[4]Clavien PA, Barkun J, de Oliveira ML, Vauthey JN, Dindo D, Schulick RD, de Santibañes E, Pekolj J, Slankamenac K, Bassi C, Graf R, Vonlanthen R, Padbury R, Cameron JL, Makuuchi M. *The Clavien-Dindo classification of surgical complications: five-year experience.* Ann Surg. 2009;250:187-96.
[5] Mayhew D, Mendonca V, Murthy BVS. *A review of ASA physical status - historical perspectives and modern developments.* Anaesthesia. 2019;74:373–379.
[6] Harryman C, Plymale MA, Stearns E, Davenport DL, Chang W, Roth JS. *Enhanced value with implementation of an ERAS protocol for ventral hernia repair.* Surg Endosc. 2020;34:3949-55.
[7] Saleh S, Plymale MA, Davenport DL, Roth JS. *Risk-Assessment Score and Patient Optimization as Cost Predictors for Ventral Hernia Repair.* J Am Coll Surg. 2018;226:540-6.
[8] Svane JK, Chiou ST, Groene O, Kalvachova M, Brkić MZ, Fukuba I, Härm T, Farkas J, Ang Y, Andersen MØ, Tønnesen H. *A WHO-HPH operational program versus usual routines for implementing clinical health promotion: an RCT in health promoting hospitals (HPH)*. Implement Sci. 2018;13:153.
[9]Tønnesen H, Nielsen PR, Lauritzen JB, Moller AM. *Smoking and alcohol intervention before surgery: evidence for best practice.* Br J Anaesth. 2009; 102:297–306.
[10] Weimann A, Braga M, Carli F, Higashiguchi T, Hübner M, Klek S, Laviano A, Ljungqvist O, Lobo DN, Martindale RG, Waitzberg D, Bischoff SC, Singer P. *ESPEN practical guideline: Clinical nutrition in surgery.* Clin Nutr. 2021;40:4745-61.
[11] Maibom SL, Røder MA, Poulsen AM, Thind PO, Salling ML, Salling LN, Kehlet H, Brasso K, Joensen UN. *Morbidity and Days Alive and Out of Hospital Within 90 Days Following Radical Cystectomy for Bladder Cancer.* Eur Urol Open Sci. 2021;28:1-8.
[12] Pathak RA, Wilson RRA, Craven TE, Matz E, Hemal AK. *The role of body mass index on quality indicators following minimally-invasive radical prostatectomy.* Investig Clin Urol. 2021;62:290-7.
[13] van Rooijen S, Carli F, Dalton S, Thomas G, Bojesen R, Le Guen M, Barizien N, Awasthi R, Minnella E, Beijer S, Martínez-Palli G, van Lieshout R, Gögenur I, Feo C, Johansen C, Scheede-Bergdahl C, Roumen R, Schep G, Slooter G. *Multimodal prehabilitation in colorectal cancer patients to improve functional capacity and reduce postoperative complications: the first international randomized controlled trial for multimodal prehabilitation.* BMC Cancer. 2019;19:98.
[14] Fried LP, Tangen CM, Walston J, Newman AB, Hirsch C, Gottdiener J, Seeman T, Tracy R, Kop WJ, Burke G, McBurnie MA; Cardiovascular Health Study Collaborative Research Group. Frailty in older adults: evidence for a phenotype. J Gerontol A Biol Sci Med Sci. 2001 Mar;56(3):M146-56. doi: 10.1093/gerona/56.3.m146.
[15] Lammers F, Zacharias N, Borchers F, Mörgeli R, Spies CD, Winterer G. *Functional connectivity of the supplementary motor network is associated with Fried's modified frailty score in older adults.* J Gerontol A. 2020;75:2239-48.
[16] Best MJ, Buller LT, Gosthe RG, Klika AK, Barsoum WK. *Alcohol Misuse is an Independent Risk Factor for Poorer Postoperative Outcomes Following Primary Total Hip and Total Knee Arthroplasty. (A cohort of 8,372,232 patients).* J Arthroplasty. 2015;30:1293-8.
[17] Eliasen M, Grønkjær M, Skov-Ettrup LS, Mikkelsen SS, Becker U, Tolstrup JS, Flensborg-Madsen T. *Preoperative alcohol consumption and postoperative complications: a systematic review and meta-analysis.* Ann Surg. 2013;258:930-42.
[18] Grønkjær M, Eliasen M, Skov-Ettrup LS, Tolstrup JS, Christiansen AH, Mikkelsen SS, Becker U, Flensborg-Madsen T. *Preoperative smoking status and postoperative complications: a systematic review and meta-analysis.* Ann Surg. 2014;259:52-71.
[19] Bohlin KS, Ankardal M, Stjerndahl JH, Lindkvist H, Milsom I. *Influence of the modifiable life-style factors body mass index and smoking on the outcome of hysterectomy.* Acta Obstet Gynecol Scand. 2016;95:65-73.
[20] Nilsson H, Angerås U, Bock D, Börjesson M, Onerup A, Fagevik Olsen M, Gellerstedt M, Haglind E, Angenete E. *Is preoperative physical activity related to post-surgery recovery? A cohort study of patients with breast cancer.* BMJ Open. 2016;6:e007997.
[21] Jensen BT, Lauridsen SV, Jensen JB. *Prehabilitation for major abdominal urologic oncology surgery.* Curr Opin Urol. 2018;28:243–50.

[22] Tønnesen H, Lydom LN, Joensen UN, Egerod I, Pappot H, Lauridsen SV. *STRONG for Surgery & Strong for Life - against all odds: intensive prehabilitation including smoking, nutrition, alcohol, and physical activity for risk reduction in cancer surgery - a protocol for an RCT with nested interview study (STRONG-Cancer).* Trials. 2022;23:333.
[23] Egholm JWM, Pedersen B, Møller AM, Adami J, Juhl CB, Tønnesen H. *Perioperative alcohol cessation intervention for postoperative complications.* Cochrane Database Syst Rev. 2014;2014(3):CD002294.
[24] Thomsen T, Villebro N, Møller AM. *Interventions for preoperative smoking cessation.* Cochrane Database Syst Rev. 2014;2014(3):CD002294.
[25] Kalarchian MA, Marcus MD, Courcoulas AP, Cheng Y, Levine MD. *Preoperative lifestyle intervention in bariatric surgery: a randomized clinical trial.* Surg Obes Relat Dis. 2016;12:180-7.
[26] Kendall F, Oliveira J, Peleteiro B, Pinho P, Bastos PT. *Inspiratory muscle training is effective to reduce postoperative pulmonary complications and length of hospital stay: a systematic review and meta-analysis.* Disabil Rehabil. 2018;40:864–82.
[27] Lobo DN, Gianotti L, Adiamah A, Barazzoni R, Deutz NEP, Dhatariya K, Greenhaff PL, Hiesmayr M, Hjort Jakobsen D, Klek S, Krznaric Z, Ljungqvist O, McMillan DC, Rollins KE, Panisic Sekeljic M, Skipworth RJE, Stanga Z, Stockley A, Stockley R, Weimann A. *Perioperative nutrition: Recommendations from the ESPEN expert group.* Clin Nutr. 2020;39:3211-27.
[28] Falz R, Bischoff C, Thieme R, Lässing J, Mehdorn M, Stelzner S, Busse M, Gockel I. *Effects and duration of exercise-based prehabilitation in surgical therapy of colon and rectal cancer: a systematic review and meta-analysis.* J Cancer Res Clin Oncol. 2022;148:2187-213.
[29] Jensen BT, Petersen AK, Jensen JB, Laustsen S, Borre M. *Efficacy of a multiprofessional rehabilitation programme in radical cystectomy pathways: a prospective randomized controlled trial.* Scand J Urol. 2015;49:133–41.
[30] Nielsen PR, Jorgensen LD, Dahl B, Pedersen T, Tonnesen H. *Prehabilitation and early rehabilitation after spinal surgery: randomized clinical trial. Clin Rehabil. 2010;24:137-48.*
[31] Assouline B, Cools E, Schorer R, Kayser B, Elia N, Licker M. *Preoperative Exercise Training to Prevent Postoperative Pulmonary Complications in Adults Undergoing Major Surgery. A Systematic Review and Meta-analysis with Trial Sequential Analysis.* Ann Am Thorac Soc. 2021;18:678-88.
[32] Carli F, Bousquet-Dion G, Awasthi R, Elsherbini N, Liberman S, Boutros M, Stein B, Charlebois P, Ghitulescu G, Morin N, Jagoe T, Scheede-Bergdahl C, Minnella EM, Fiore JF Jr. *Effect of Multimodal Prehabilitation vs Postoperative Rehabilitation on 30-Day Postoperative Complications for Frail Patients Undergoing Resection of Colorectal Cancer: A Randomized Clinical Trial.* JAMA Surg. 2020;155:233-42. doi: 10.1001/jamasurg.2019.5474. [Erratum not related to outcomes in JAMA Surg. 2020;155:269]
[33] McIsaac DI, Hladkowicz E, Bryson GL, Forster AJ, Gagne S, Huang A, Lalu M, Lavallée LT, Moloo H, Nantel J, Power B, Scheede-Bergdahl C, van Walraven C, McCartney CJL, Taljaard M. *Home-based prehabilitation with exercise to improve postoperative recovery for older adults with frailty having cancer surgery: the PREHAB randomised clinical trial*. Br J Anaesth. 2022;129:41-8
[34] Ommundsen N, Wyller TB, Nesbakken A, Bakka AO, Jordhøy MS, Skovlund E, Rostoft S. *Preoperative geriatric assessment and tailored interventions in frail older patients with colorectal cancer: a randomized controlled trial.* Colorectal Dis. 2018; 20:16-25.
[35] Hempenius L, Slaets JP, van Asselt D, de Bock GH, Wiggers T, van Leeuwen BL. *Outcomes of a Geriatric Liaison Intervention to Prevent the Development of Postoperative Delirium in Frail Elderly Cancer Patients: Report on a Multicentre, Randomized, Controlled Trial*. PLoS One. 2013;8:e64834.
[36] Smith NA, Martin G, Marginson B. *Preoperative assessment and prehabilitation in patients with obesity undergoing non-bariatric surgery: A systematic review.* J Clin Anesth. 2022;78:110676.
[37] Liljensøe A, Laursen JO, Bliddal H, Søballe K, Mechlenburg I. *Weight Loss Intervention Before Total Knee Replacement: A 12-Month Randomized Controlled Trial.* Scand J Surg. 2021;110:3-12.
[38] Jensen KK, East B, Jisova B, Cano ML, Cavallaro G, Jørgensen LN, Rodrigues V, Stabilini C, Wouters D, Berrevoet F. *The European Hernia Society Prehabilitation Project: a systematic review of patient prehabilitation prior to ventral hernia surgery.* Hernia. 2022;26:715-26.
[39] Lauridsen SV, Thomsen T, Kaldan G, Lydom LN, Tønnesen H. *Smoking and alcohol cessation intervention in relation to radical cystectomy: a qualitative study of cancer patients' experiences.* BMC Cancer. 2017;17:793.
[40] Lauridsen SV, Thomsen T, Jensen JB, Kallemose T, Schmidt Behrend M, Steffensen K, Poulsen AM, Jacobsen A, Walther L, Isaksson A, Thind P, Tønnesen H. *Effect of a Smoking and Alcohol Cessation Intervention Initiated Shortly Before Radical Cystectomy-the STOP-OP Study: A Randomised Clinical Trial*. Eur Urol Focus. 2022:S2405-4569(22)00050-5. Online ahead of print.
[41] Neumann T, Rasmussen M, Ghith N, Heitmann BL, Tønnesen H. *The Gold Standard Programme: smoking cessation interventions for disadvantaged smokers are effective in a real-life setting*. Tob Control. 2013;22(6):e9.
[42] Merzaai B, Tonnesen H, Rasmussen M, Lauridsen SV. *Perioperative Alcohol and Smoking Cessation Intervention: Impact on Other Lifestyles.* Semin Oncol Nurs. 2021;37:151116.
[43] Stein M, Cassara EL. *Preoperative pulmonary evaluation and therapy for surgery patients.* JAMA 1970; 211:787-90.
[44] Gram-Hanssen A, Christophersen C, Rosenberg J. *Results from patient-reported outcome measures are inconsistently reported in inguinal hernia trials: a systematic review.* Hernia. 2022;26:687-99.
[45]Gram-Hanssen A, Jessen ML, Christophersen C, Zetner D, Rosenberg J. *Trends in the use of patient-reported outcome measures for inguinal hernia repair: a quantitative systematic review.* Hernia. 2021;25:1111-20.
[46] https://www.herniedatabasen.dk/eng (assessed July 31st, 2022)
[47] Sørensen LT, Hemmingsen U, Jørgensen T. *Strategies of smoking cessation intervention before hernia surgery—effect on perioperative smoking behavior.* Hernia 2007;11 327–33.

[48] Lindström D, Sadr Azodi O, Wladis A, Tønnesen H, Linder S, Nåsell H, Ponzer S, Adami J. *Effects of a perioperative smoking cessation intervention on postoperative complications: a randomized trial.* Ann Surg. 2008;248:739-45.
[49] Liang MK, Bernardi K, Holihan JL, Cherla DV, Escamilla R, Lew DF, Berger DH, Ko TC, Kao LS. *Modifying Risks in Ventral Hernia Patients with Prehabilitation: A Randomized Controlled Trial.* Ann Surg. 2018;268:674-80.
[50] Bernardi K, Olavarria OA, Dhanani NH, Lyons N, Holihan JL. Cherla DV, Berger DH, Ko TC, Kao LS, Liang MK. *Two-year Outcomes of Prehabilitation Among Obese Patients with Ventral Hernias: A Randomized Controlled Trial*. Ann Surg 2022; 275:288-94.
[51] Schwilk B, Bothner U, Schraag S, Georgieff M. *Perioperative respiratory events in smokers and nonsmokers undergoing general anaesthesia.* Acta Anaesthesiol Scand. 1997;41:348-55.
[52] Park H, de Virgilio C, Kim DY, Shover AL, Moazzez A. *Effects of smoking and different BMI cutoff points on surgical site infection after elective open ventral hernia repair.* Hernia. 2021;25:337-43.
[53] Sorensen LT, Friis E, Jorgensen T, Vennits B, Andersen BR, Rasmussen GI, Kjaergaard J. *Smoking is a risk factor for recurrence of groin hernia.* World J Surg. 2002;26:397-400.
[54] Parker SG, Mallett S, Quinn L, Wood CPJ, Boulton RW, Jamshaid S, Erotocritou M, Gowda S, Collier W, Plumb AAO, Windsor ACJ, Archer L, Halligan S. *Identifying predictors of ventral hernia recurrence: systematic review and meta-analysis.* BJS Open. 2021;5:zraa071. [Erratum - without relation to the results - in BJS Open. 2021:7;5]
[55] Tønnesen H, Lydom LN, Joensen UN, Egerod I, Pappot H, Lauridsen SV. *STRONG for Surgery & Strong for Life - against all odds: intensive prehabilitation including smoking, nutrition, alcohol, and physical activity for risk reduction in cancer surgery - a protocol for an RCT with nested interview study (STRONG-Cancer).* Trials. 2022 Apr 21;23(1):333.
[56] Tønnesen H (Editor), Phrochaska JO, Chiou ST, Schroeder TS, Larsen ML, Høst H, Gentilello LM, Damsgaard EMS, Spies CD, Berger H, Groene O, Clark JG, Trychin S, Fawkes S, Kildedal KCB, Jensen TB. *Engage in the process of change – facts and methods.* 2012 Copenhagen, WHO Regional Office for Europe. ISBN (Print) 978-87-9. Https://portal.research.lu.se/en/publications/engage-in-the-process-of-change-facts-and-methods94329-0-5. (assessed July 31st, 2022)
[57] Malterud K. *Systematic text condensation: A strategy for qualitative analysis.* Scand J Public Health. 2012;40:795–805.
[58] Slankamenac K, Graf R, Barkun J, Puhan MA, Clavien PA. *The comprehensive complication index: a novel continuous scale to measure surgical morbidity*. Ann Surg. 2013;258:1-7.
[59] Gillis C, Ljungqvist O, Carli F. *Prehabilitation, enhanced recovery after surgery, or both? A narrative review.* Br J Anaesth. 2022;128:434-48. [Erratum of figure 2 in: Br J Anaesth. 2022;128:1061]
[60] Birkelbach O, Mörgeli R, Spies C, Olbert M, Weiss B, Brauner M, Neuner B, Francis RCE, Treskatsch S, Balzer F. *Routine frailty assessment predicts postoperative complications in elderly patients across surgical disciplines - a retrospective observational study.* BMC Anesthesiol. 2019;19:204.
[61] Schaller SJ, Kiselev J, Loidl V, Quentin W, Schmidt K, Mörgeli R, Rombey T, Busse R, Mansmann U, Spies C; PRAEP-GO consortium, PRAEP-GO investigators. Prehabilitation of elderly frail or pre-frail patients prior to elective surgery (PRAEP-GO): study protocol for a randomized, controlled, outcome assessor-blinded trial. Trials. 2022 Jun 6;23(1):468. doi: 10.1186/s13063-022-06401-x. Erratum in: Trials. 2023 Feb 14;24(1):111. PMID: 35668532; PMCID: PMC9167908..
[62] Zigmond AS, Snaith RP. *The hospital anxiety and depression scale.* Acta Psychiatr Scand. 1983;67:361-70.
[63]Badia JM, Casey AL, Petrosillo N, Hudson PM, Mitchell SA, Crosby C. Impact of surgical site infection on healthcare costs and patient outcomes: a systematic review in six European countries. J Hosp Infect. 2017 May;96(1):1-15. doi: 10.1016/j.jhin.2017.03.004.
[64] Rasmussen M, Fernández E, Tønnesen H. *Effectiveness of the Gold Standard Programme compared with other smoking cessation interventions in Denmark: a cohort study.* BMJ Open. 2017;7:e013553.
[65] Egholm JWM, Pedersen B, Oppedal K, Madsen BL, Lauritzen JB, Rasmussen M, Helander A, Adami J, Tønnesen H. *Minor effect of patient education for alcohol cessation intervention on outcomes after acute fracture surgery: a randomized trial of 70 patients.* Acta Orthop. 2022;93:424-31.
[66] Rasmussen M, Hovhannisyan K, Adami J, Tønnesen H. *Characteristics of Patients in Treatment for Alcohol and Drug Addiction Who Succeed in Changing Smoking, Weight, and Physical Activity: A Secondary Analysis of an RCT on Combined Lifestyle Interventions.* Eur Addict Res. 2021;27:123-30..
[67] Møller AM, Villebro N, Pedersen T, Tønnesen H. *Effect of preoperative smoking intervention on postoperative complications: a randomised clinical trial.* Lancet. 2002;359:114-7.
[68] Heatherton TF, Kozlowski LT, Frecker RC, Fagerström KO. *The Fagerström test for nicotine dependence: a revision of the Fagerström tolerance questionnaire.* Br J Addict 1991;86:1119–27.
[69] https://www.regionh.dk/til-fagfolk/Forskning-og-innovation/Teknologisk-infrastruktur-og-it/REDCAP/Sider/REDCap-kvalitetsh%C3%A5ndbog.aspx (assessed July 31st, 2022)
